# Parallel analysis of miRNAs and mRNAs suggests distinct regulatory networks in *Crassostrea gigas* infected by Ostreid herpesvirus 1

**DOI:** 10.1186/s12864-020-07026-7

**Published:** 2020-09-10

**Authors:** Umberto Rosani, Miriam Abbadi, Timothy Green, Chang-Ming Bai, Edoardo Turolla, Giuseppe Arcangeli, K. Mathias Wegner, Paola Venier

**Affiliations:** 1 Department of Biology, University of Padova, 35121 Padova, Italy; 2 Coastal Ecology Section, AWI - Alfred Wegener Institute Helmholtz Centre for Polar and Marine Research, Wadden Sea Station Sylt, 25992 List, Germany; 3 Istituto Zooprofilattico delle Venezie, Legnaro, Italy; 4 Centre for Shellfish Research & Department of Fisheries and Aquaculture, Vancouver Island University, Nanaimo, BC V9R 5S5 Canada; 5 Yellow Sea Fisheries Research Institute, Chinese Academy of Fishery Sciences, Qingdao, 266071 China; 6 CRIM Laboratory - Delta Institute, Goro, Ferrara, Italy

**Keywords:** Oyster, miRNA, OsHV-1, ADAR, *C. gigas*, miRNAome

## Abstract

**Background:**

Since 2008, the aquaculture production of *Crassostrea gigas* was heavily affected by mass mortalities associated to Ostreid herpesvirus 1 (OsHV-1) microvariants worldwide. Transcriptomic studies revealed the major antiviral pathways of the oyster immune response while other findings suggested that also small non-coding RNAs (sncRNA) such as microRNAs might act as key regulators of the oyster response against OsHV-1. To explore the explicit connection between small non-coding and protein-coding transcripts, we performed paired whole transcriptome analysis of sncRNA and messenger RNA (mRNA) in six oysters selected for different intensities of OsHV-1 infection.

**Results:**

The mRNA profiles of the naturally infected oysters were mostly governed by the transcriptional activity of OsHV-1, with several differentially expressed genes mapping to the interferon, toll, apoptosis, and pro-PO pathways. In contrast, miRNA profiles suggested more complex regulatory mechanisms, with 15 differentially expressed miRNAs (DE-miRNA) pointing to a possible modulation of the host response during OsHV-1 infection. We predicted 68 interactions between DE-miRNAs and oyster 3′-UTRs, but only few of them involved antiviral genes. The sncRNA reads assigned to OsHV-1 rather resembled mRNA degradation products, suggesting the absence of genuine viral miRNAs.

**Conclusions:**

We provided data describing the miRNAome during OsHV-1 infection in *C. gigas*. This information can be used to understand the role of miRNAs in healthy and diseased oysters, to identify new targets for functional studies and, eventually to disentangle cause and effect relationships during viral infections in marine mollusks.

## Background

Mollusk aquaculture is regarded as the fastest growing food production sector and plays a vital role in solving the problem of feeding future human generations [1, 2]. However, the recurrence of infectious disease as well as intensive mono-specific farming of bivalve species threaten this production sector [3, 4]. Global oyster production has particularly suffered from Pacific oyster mortality syndrome (POMS), a deadly disease mainly caused by Ostreid herpesvirus 1 (OsHV-1) infection [5]. OsHV-1 is one of the two dsDNA viruses of the *Malacoherpesviridae* family [6], that can infect oysters and other bivalve species [7–10], whereas the other, Haliotid herpesvirus 1 (HaHV-1), causes a disease called abalone viral ganglioneuritis in gastropod species [11, 12]. Different genomic variants of these two viruses have been identified in several disease outbreaks and the virus presence in healthy individuals or non-susceptible species, as reservoirs for viral particles, makes virus eradication almost impossible. Hence, the understanding of virus life strategies and host-pathogen interactions in different host species is essential for the prevention and management of mass mortalities [13]. At present, the lack of mollusk cell lines [13] hampers the in vitro propagation of malacoherpesviruses, even if cultured hemocytes have been recently proposed as a tool to propagate the virus and to perform small-scale infection trials [14, 15]. The available knowledge on *Malacoherpesviridae* biology refers to in vivo infections, either based on laboratory trials or on the rare cases of naturally infected bivalves and, mostly, by high-throughput (HT) sequencing [16]. Notably, recent studies suggest that the weakening of immune defenses consequent to OsHV-1 infection is likely to induce critical changes in the oyster-associated microbiota and, hence, fatal secondary infections [17, 18]. The transcriptional host response has been characterized in *Crassostrea gigas* and *Scapharca broughtonii* infected with OsHV-1 [19–21] and, more recently, for *Haliotis spp*. infected with HaHV-1 [22, 23], revealing distinct antiviral responses in the different virus-host combinations. Infected *C. gigas* mainly activated the interferon pathway to produce antiviral molecules known as interferon stimulated genes (e.g., viperin) [19, 21, 24]. Differently, *S. broughtonii* infected with the same virus mostly modulated apoptosis-related genes similar to *H. diversicolor* infected with HaHV-1 [20, 22]. These mechanistic differences show that we still lack a comprehensive understanding of the transcriptional response to viral infections in mollusks.

Besides the expression of coding RNAs, non-coding RNAs (ncRNAs) such as small and long non-coding RNAs (sncRNAs, lncRNAs) can play an important role during viral infections [25]. ncRNAs are known as post-transcriptional regulators [26], and they can participate in virus-host interactions through a variety of mechanisms [27, 28]. Among the ncRNAs, miRNAs are the most frequently studied due to their involvement in various diseases and their potential as diagnostic and prognostic biomarkers [29]. Mature miRNAs are short sequences (20–23 nt), which originate from stem-loop precursor RNAs (pre-miRNAs) and act as post-transcriptional repressors by targeting untranslated regions (UTRs) of messenger RNAs (mRNAs) in biological processes such as immunity, development, cell behavior and host-microorganism interactions [30, 31]. After the first discovery of *lin-14* in *Caenorhabditis elegans* [32], miRNAs have been identified in many animals, plants, and viruses, suggesting independent miRNA origins throughout the major evolutionary lineages [26]. Virus-encoded miRNAs have been reported in vertebrate herpesviruses, for instance in *Epstein-Barr virus* [33] where they perform immuno-modulatory functions [34]. Regarding invertebrate viruses, miRNAs have only been reported in the *White Spot Syndrome Virus* (WSSV) [35]. WSSV miRNAs target the JAK-STAT signaling pathway to interfere with the immune system of the shrimp [36], and their sequences are actively edited by host enzymes, like the adenosine deaminase acting on dsRNAs (ADAR-1) [37].

In bivalves, only a few studies investigated the presence and role of miRNAs in relation to biomineralization [38–40] and neuro-immunity [41, 42], whereas a single study investigated the roles of miRNAs in scallops (*Chlamys farreri*) infected by an OsHV-1 variant [43] (the related data are not public). Therefore, we have examined the relationship between coding and non-coding RNAs by parallel HT-sequencing of mRNA and sncRNA in oysters naturally infected by OsHV-1. Based on the coupled transcriptomic landscapes of mRNAs and sncRNAs we add an additional facet to the characterization of the molecular actions and counteractions in OsHV-1 infections of oysters.

## Results

### OsHV-1 infection in field-exposed oysters

**Fig. 1:**
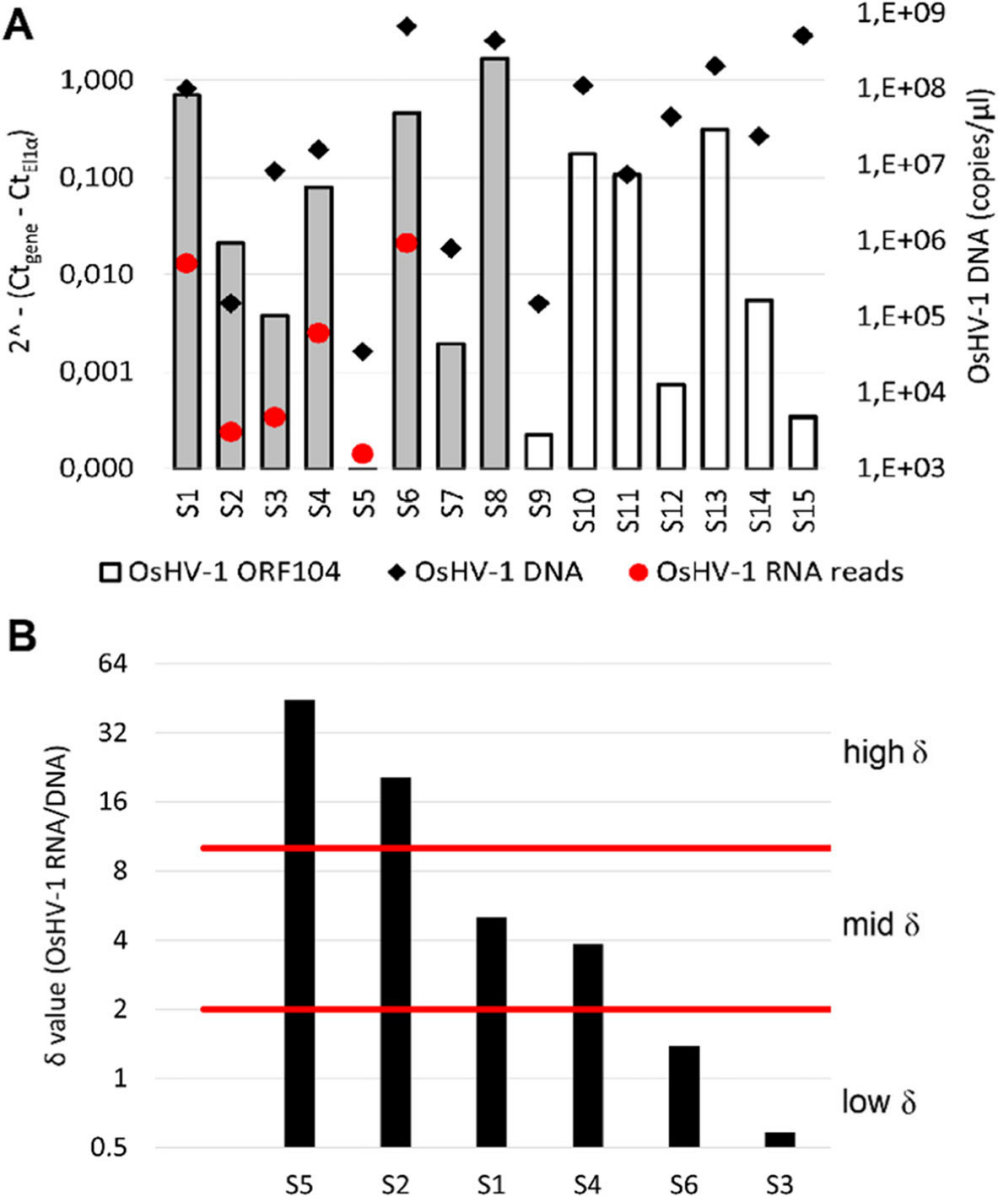
OsHV-1 RNA and DNA levels in the gill tissue of 15 OsHV-1-positive oysters (*C. gigas*, S1-S15) sampled in the Goro lagoon, Italy. **a.** Quantification of OsHV-1 transcription by RT-qPCR (bars, left axis) and OsHV-1 DNA loads (black diamonds, right axis). The OsHV-1 ORF104 transcript levels normalized to the expression of the *C. gigas* housekeeping gene *elongation factor 1-apha,* was considered as a proxy of the viral transcriptional activity. OsHV-1 DNA content was measured as DNA copy number per μl. Grey bars indicate the six samples selected for sncRNA and mRNA HT-sequencing. In these six samples, red dots represent the number of RNA reads mapping to the OsHV-1 genome. The samples denoted by grey bars were selected for RT-qPCR analysis. **b.** Subdivision of samples S1-S6 based on the ratio of OsHV-1 RNA over DNA (δ value) grouping pairs of samples into low, mid, or high δ samples

We detected variable amounts of OsHV-1 DNA in 15 oysters collected from Goro lagoon on May 17, 2016 (Fig. 1a). In 8 of the 15 samples, the expression levels of OsHV-1 ORF104, a proxy for total viral transcription, were higher than 1% of the expression level of the host housekeeping gene Elongation factor-1-alpha (Fig. 1a). We further selected 6 oyster gill samples, representative of a range of OsHV-1 DNA and RNA levels, to perform parallel high-throughput sequencing of sncRNAs and (poly(A)-tail selected) mRNAs. In detail, we sequenced two samples (S2 and S5) with high viral RNA: DNA ratios (δ) and low absolute amounts of OsHV-1 DNA, two samples (S1 and S4), with an intermediate δ value and intermediate to high levels of OsHV-1 DNA and two samples (S3 and S6) with the lowest δ values and intermediate to high levels of OsHV1 DNA (Fig. 1b, Table 1). Taking into consideration the presence of OsHV-1 DNA, high and intermediate δ values suggest active OsHV-1 in the early phases of the infection, whereas low δ values suggest a limited transcription of abundantly present OsHV-1. This latter situation could be interpreted as a late infection stage. Overall, the high-throughput sequencing of the six samples yielded 94.2 M sncRNA reads (size range: 18–40 nt) and 380 M mRNA reads (Table 1).

**Table 1:** HT-sequencing results showing the amounts of mRNA and sncRNA reads in the oyster samples. Sample classification was based on δ values. Amount of OsHV-1 DNA (copies per μl), number of clean reads and number of oyster and OsHV-1 reads are reported for each library. For the sample S6, details of the ribo-depleted library are also reported

Sample ID	Sample classification (Fig. 1b)	OsHV-1 DNA [copies/μl]	Library type	Clean reads [M]	***C. gigas*** reads [%]	OsHV-1 reads [%]
S1	Mid	1 × 10^8^	mRNA	53.51	91.6	0.84
			sncRNA	13.61	96.8	0.07
S2	High	1.5 × 10^5^	mRNA	64.92	91.5	< 0.01
			sncRNA	13.66	96.7	0.04
S3	Low	8.3 × 10^6^	mRNA	59.17	90.4	< 0.01
			sncRNA	26.79	95.8	0.05
S4	Mid	1.6 × 10^7^	mRNA	46.41	91.6	0.12
			sncRNA	13.98	97.1	0.03
S5	High	3.5 × 10^4^	mRNA	50.53	92.4	< 0.01
			sncRNA	12.91	95.9	0.03
S6	Low	6.7 × 10^8^	mRNA	51.98	87.6	1.64
			sncRNA	13.33	90.7	0.07
			total RNA	54.13	64.8	1.89

Library types: *mRNA* RNA-seq libraries obtained by selecting the polyA+ RNAs, *sncRNA* small non-coding RNA libraries; total RNA, RNA-seq library obtained by ribosomal rRNA depletion

### miRNA expression during OsHV-1 infection

**Fig. 2:**
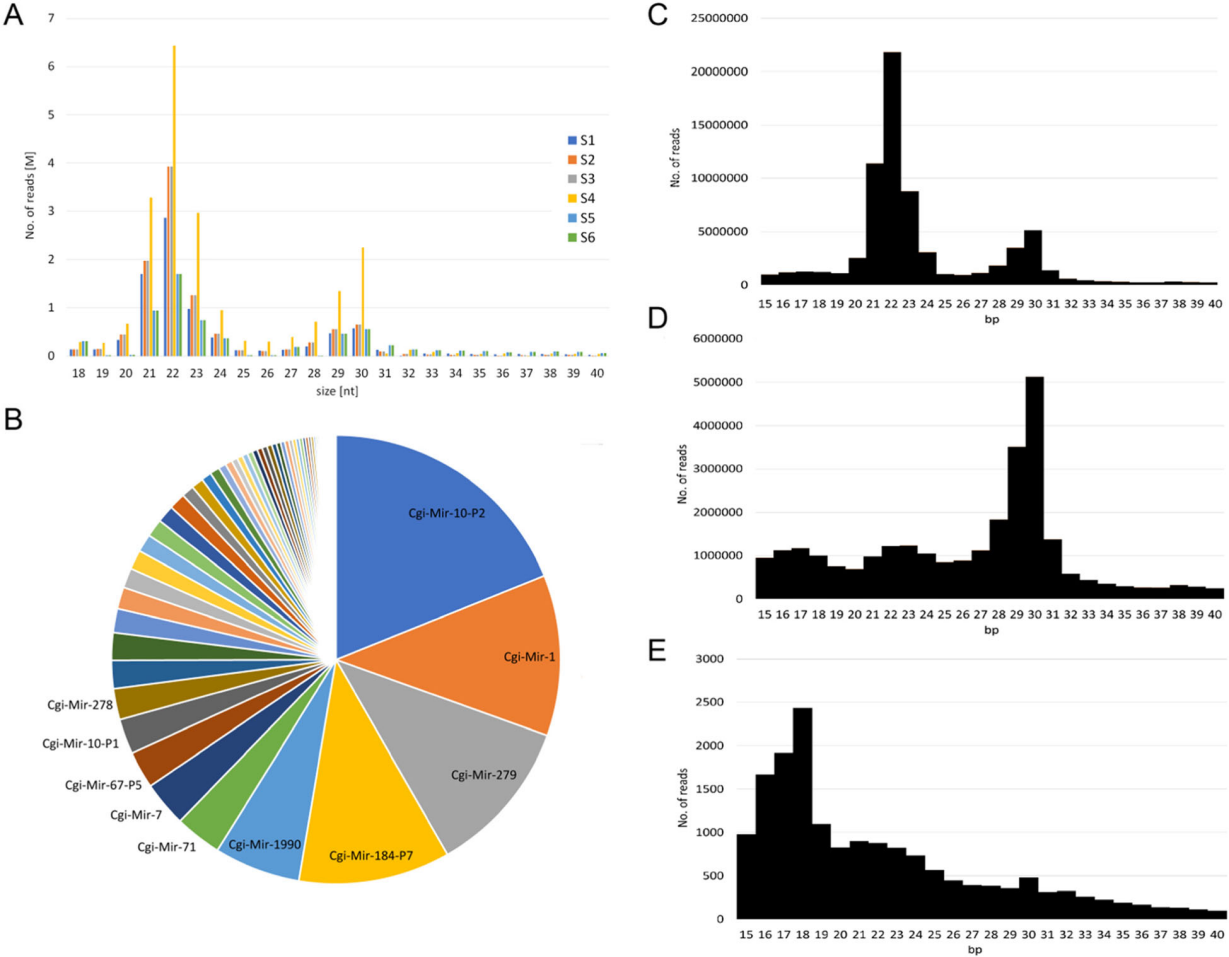
sncRNA analysis. **a.** Size distribution of the sncRNA reads in the six libraries (size range: 18–40 nucleotides). **b**. Cumulative (S1-S6) expression of *C. gigas* miRNAs classified according to MirGeneDB v.2.0 (57 miRNAs contributing to less than 0.1% to the global expression were removed; data are available in Additional file 1). **c.** Cumulative size distribution of the sncRNA reads mapping to the oyster miRNA precursors. **D, E**. The sncRNA reads not matching to oyster miRNAs were mapped to the oyster (**D**) or to the OSHV-1 genome (**E**)

The size distributions of the sncRNA reads in the six samples showed a clear 20–22 nt peak, typical of the presence of miRNAs, with a secondary peak around 30 nt, possibly related to the presence of piwi-interacting RNAs (Fig. 2a). The percentage of the sncRNA reads mapping to the oyster genome ranged from 90.7 to 97.1%, whereas only a small fraction of the reads mapped to the OsHV-1 genome (0.03–0.07%, Table 1). Overall, 46% of the sncRNA reads mapped to the 151 *C. gigas* miRNA precursors retrieved from MirGeneDB v.2.0 [44], showing a clear 22-nt peak (Fig. 2c). The reads not mapping to the 151 oyster miRNA precursors mostly found a match in the *C. gigas* genome, showing a clear 29-nt peak (Fig. 2d), while the reads matching to the OsHV-1 genome showed shorter size (17–19 nt, Fig. 2e). We verified the presence of the minimal miRNA annotation criteria for the 151 oyster miRNA precursors, including read coverage on both miRNA arms, 5′ read homogeneity and the absence of read mapping in the surroundings of the miRNA arms [45]. Accordingly, we confirmed most of these oyster miRNA predictions with the following exceptions: i) we could not find reads mapping to the star arm for some miRNAs such as *Cgi-mir-96-P3, Cgi-mir-87, Cgi-novel-4, Cgi-novel-18* and several *Cgi-mir-184* isoforms, ii) we found equal coverage of both mature and star arms in the case of 4 miRNA precursors, and iii) we reverted the mature and star predictions because of differential coverages for other 7 miRNA precursors (*Cgi-mir-36, Cgi-mir-1992, Cgi-novel-1, 2, 8, 13* and *Cgi-novel-15*).

We could compute expression values for 132 out of 151 miRNAs (Additional file 1), although 57 of these 132 miRNAs cumulatively contributed less than 0.1% to the global expression and included most of the *Cgi-mir-184* isoforms as well as 6 out of 7 novel oyster miRNAs (*Cgi-novel-1, 2, 5, 10, 21, 22,* Additional file 1). *Cgi-*m*ir-10-P2*, *Cgi-mir-1*, *Cgi-mir-279* and *Cgi-mir-184-P7* resulted to be the most expressed miRNAs, accounting for 18.9, 11.6, 11.2 and 10.9% of the global miRNA expression, respectively (Fig. 2b and Additional file 1). Among them, *Cgi-mir-184-P7* and *Cgi-*m*ir-10-P2* displayed the most stable expression levels over the six tested samples, with a coefficient of variation of 3 and 9%, respectively. These miRNAs were tested as references for RT-qPCR analysis. Two *Cgi-mir-375* isoforms, *Cgi-mir-750*, *Cgi-mir-1175* and *Cgi-novel-19* were the most variable miRNAs among the 6 samples, with a CV > 100%. Four out of these (two *Cgi-mir-375* isoforms, *Cgi-mir-750* and *Cgi-mir-1175)* mapped in near vicinity to each other on the oyster genome. Next to this cluster, another set of neighboring miRNAs (*Cgi-mir-12* and two *Cgi-mir-216* isoforms) also showed considerable variation in their expression levels (Additional file 1).

**Fig. 3:**
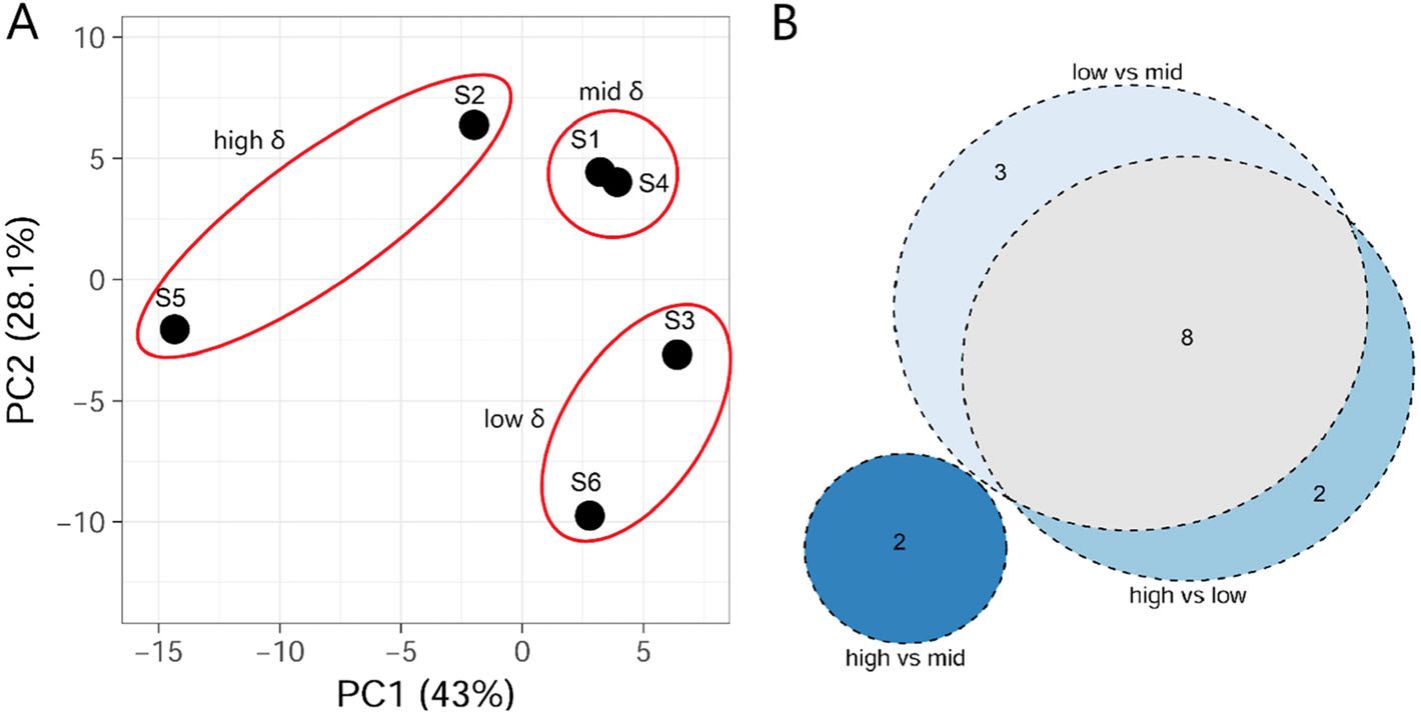
**a.** Principal Component Analysis of the miRNA expression in samples S1-S6. **b**. Venn diagram depicting the differentially expressed miRNAs in the pairwise comparisons of the samples grouped by δ values

Overall, the miRNA expression changes seemed to be influenced by OsHV-1 activity. Considering the expression of all miRNAs together in a Principal Component Analysis (PCA), we could confirm the three δ groups, with the ‘high’ group separated by axis one, and the ‘low’ group separated by axis 2 (Fig. 3a). Accordingly, we searched for differentially expressed miRNAs (DE-miRNAs) in pairwise comparisons between the groups. The strongest response could be observed in the ‘low’ group, which had 11 DE-miRNAs compared to the ‘mid’ group and 10 DE-miRNAs compared to the ‘high’ group (with 8 DE-miRNAs shared between both contrasts, Fig. 3 and Table 2). The list of the 15 DE-miRNAs included only one novel miRNA (*Cgi-novel-19*). *Cgi-novel-10* was expressed almost exclusively in the “low δ “samples S3 and S6, but high variation in expression levels yielded no statistically significance when applying the same criteria as for the other DE-miRNAs.

**Table 2:** Differentially expressed miRNAs in pairwise comparisons among samples S1-S6. Table heading: miRNA ID, the comparison in which a specific miRNA resulted to be a differentially expressed and miRNA expression values per samples are reported for the 15 DE-miRNAs

miRNA ID	DE-miRNA in comparison	High δ		Mid δ		Low δ	
		S5	S2	S1	S4	S6	S3
*Cgi-Mir-1990*	low vs mid / high vs low	73,560.6	77,564.1	76,300.4	72,135.1	31,006.6	41,873.0
*Cgi-Mir-1985*	high vs mid	5209.6	7205.2	19,485.6	17,758.6	29,735.9	14,184.6
*Cgi-Mir-10-P5*	low vs mid	16,886.8	9023.2	9753.8	6125.4	18,234.3	15,523.1
*Cgi-Mir-216-P1*	low vs mid / high vs low	3735.5	2311.1	1932.2	3098.0	13,186.0	17,259.5
*Cgi-Mir-315-v1*	low vs mid / high vs low	3702.3	2991.5	3022.3	4002.2	9081.9	6761.7
*Cgi-Mir-92-o34*	high vs low	4628.8	3383.2	3812.6	3012.5	1954.6	1942.7
*Cgi-Mir-193-P1*	high vs mid	1285.2	1415.8	2561.8	2908.5	4394.2	2301.6
*Cgi-Mir-375-P4*	low vs mid / high vs low	1602.7	70.0	253.1	193.9	4030.3	5542.1
*Cgi-Mir-133-v2*	low vs mid	3538.9	1377.4	1355.8	1185.2	533.4	489.0
*Cgi-Mir-133-v1*	low vs mid	3537.8	1377.3	1355.2	1184.8	533.1	489.0
*Cgi-Mir-315-v2*	low vs mid / high vs low	791.0	842.1	836.6	1041.4	2615.2	1959.1
*Cgi-Mir-12*	low vs mid / high vs low	759.2	632.7	534.2	783.2	2143.5	2008.4
*Cgi-Mir-92-o31*	high vs low	1062.7	779.5	1058.3	603.7	354.8	446.9
*Cgi-Mir-1993*	low vs mid / high vs low	258.8	108.5	101.8	139.6	708.7	606.4
*Cgi-Novel-19*	low vs mid / high vs low	106.5	0.1	0.2	0.1	329.9	288.3

### Validation of miRNA expression by RT-qPCR

**Fig. 4:**
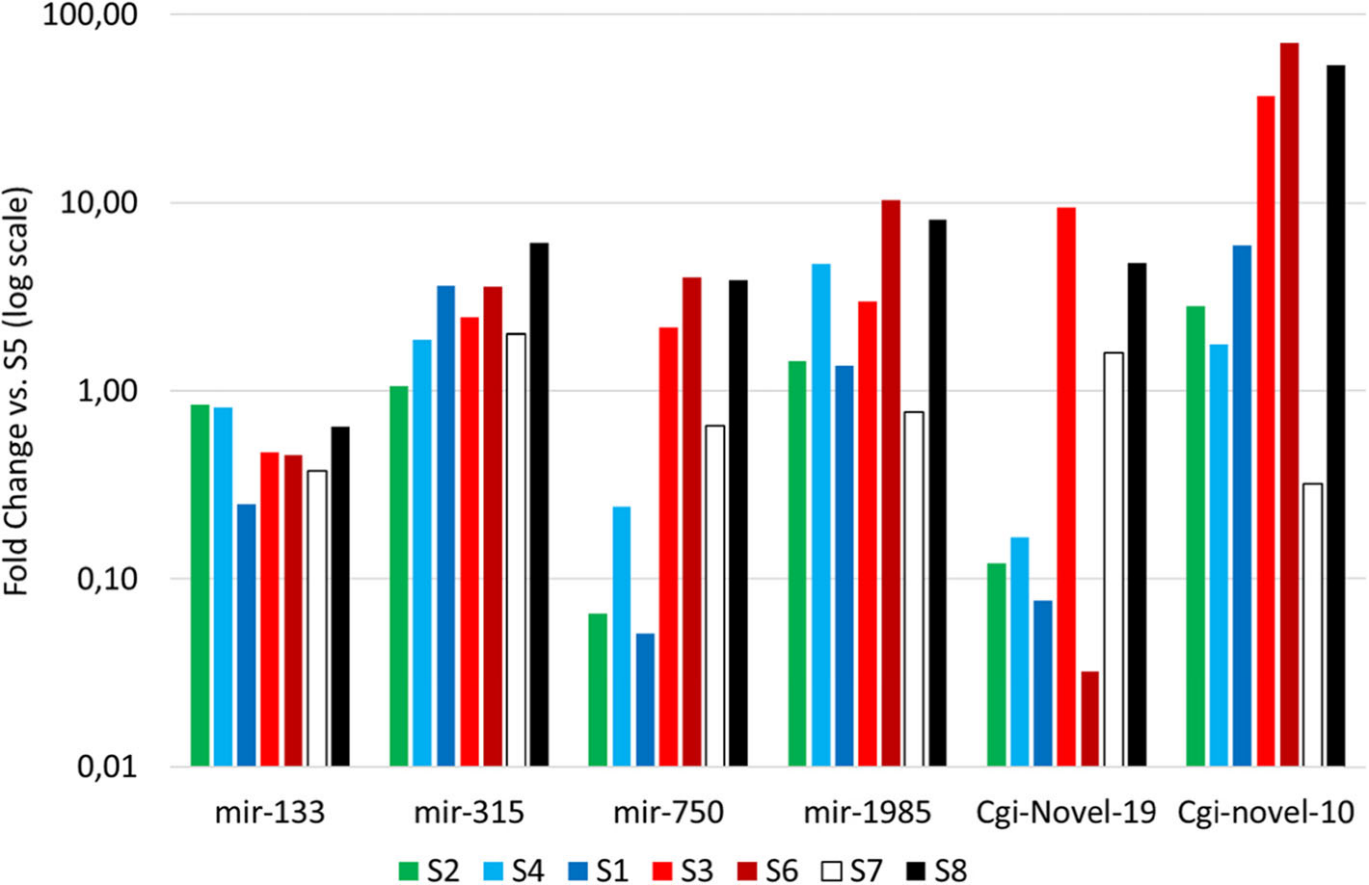
RT-qPCR analysis. The expression values of six selected oyster miRNAs over 8 samples are expressed as fold change versus the values measured in the sample S5. Samples are colored following the delta values, green for high value (S5), bluish for intermediate values (S1 and S4), reddish for low values (S3 and S6). The two additional samples (S7 and S8) for which we cannot compute a delta value are reported in white (low infection) or black (high infection)

According to overall expression patterns, we selected 8 miRNAs for expression validation by RT-qPCR using the six RNA samples used for sncRNA sequencing plus two samples selected among the 15 and representing a case of low (sample S7) and a high infection (sample S8). We selected *Cgi-mir-184-P7* as housekeeping miRNAs because of its low variation of mapped reads among samples and stability compared to the spiked RNA Sp6. *Cgi-mir-133*, *Cgi-mir-315*, *Cgi-mir-1985* and *Cgi-Novel-19* were chosen within the DE-miRNAs to cover different expression ranges. Additionally, we included two miRNAs (*Cgi-mir-750* and *Cgi-novel-10*) because of their contrasted expression patterns in sncRNA-seq data. After data normalization, the correlation between sncRNA-seq and RT-qPCR expression levels for the six samples (S1-S6) ranged from a r^2^ of 0.85 to 0.99 (Additional file 2). Following RT-qPCR, also the two additional samples showed miRNA expression values that matched the trends obtained by HTS, further supporting the link between expression of the majority of miRNAs consistently depends on the OsHV-1 infection intensity (Additional file 2). When normalizing miRNA expression values to sample S5 (one of the two samples denoted by a high δ value and characterized by the low OsHV-1 DNA load and transcription), *Cgi-mir-750* was one of the most expressed and induced miRNAs (Additional file 2 and Fig. 4). The oyster specific miRNA *Cgi-novel-10* was also highly induced in samples with comparatively higher viral activity, fitting well to the sample grouping based on δ values. In contrast, the other oyster specific miRNA *Cgi-novel-19* did not fit according to such sample grouping. *Cgi-mir-133* and *Cgi-mir-1985* and *Cgi-mir-315*, which showed intermediate expression levels (Additional file 2), were mildly induced or downregulated compared to sample S5 (Fig. 4).

### Expression of *C. gigas* coding genes during OsHV-1 infection

**Fig. 5:**
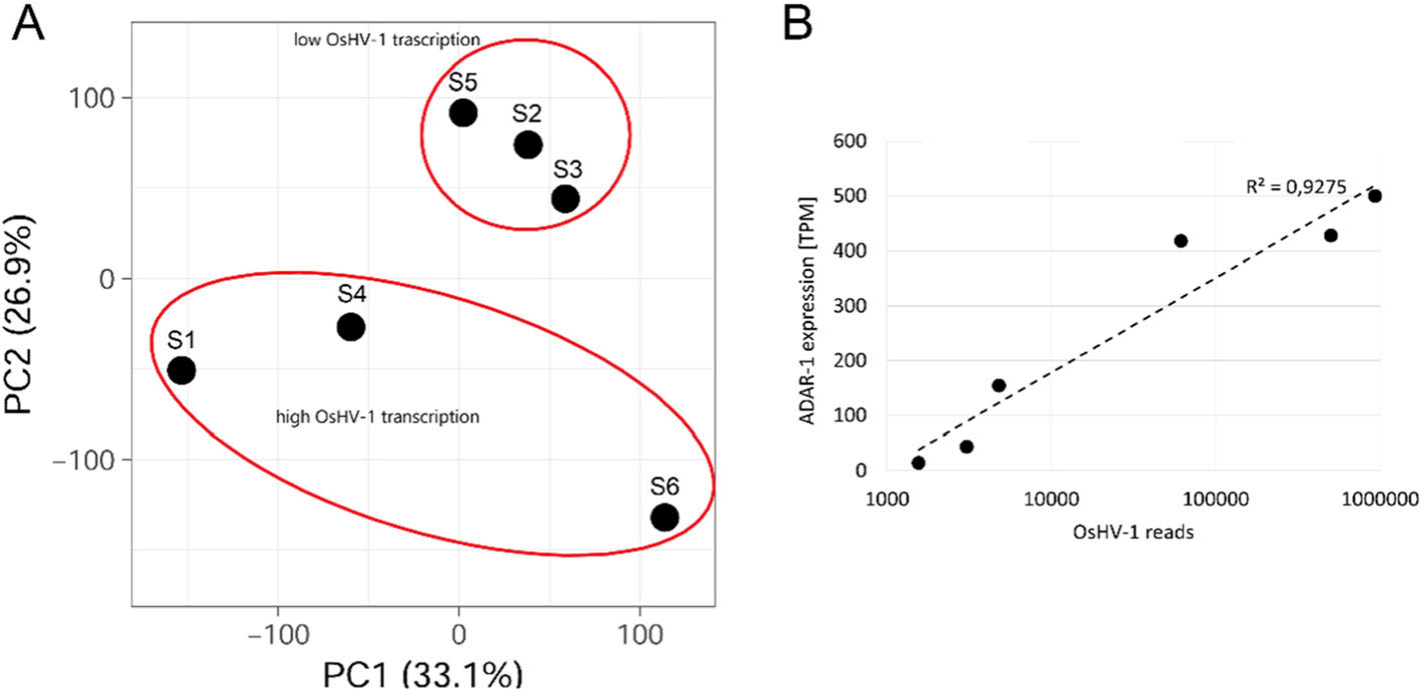
**a.** PCA analysis of samples S1-S6 based on the *C. gigas* mRNA expression profiles. The samples divided according to the presence of low or high OsHV-1 transcription levels. **b.** Correlation between the expression values of oyster ADAR-1 and the number of OsHV-1 RNA reads in the six samples

Overall, 87–92.4% of the mRNA reads mapped to the oyster genome under the applied parameters (Table 1). The PCA analysis based on the whole expression profiles outlined two groups along axis 2 (Fig. 5). These groups did not completely match the grouping of relative OsHV-1 expression (i.e. δ value), but rather corresponded to the overall OsHV-1 transcriptional activity in the samples (Fig. 1). Within the samples showing high viral transcription (S1, S4 and S6), S6 was separated along axis 1 from samples S1 and S4, while samples with low OsHV-1 transcription (S2, S3 and S5) clustered together on both axes (Fig. 5). The comparison between these two groups identified 403 DEGs, 224 up-regulated and 179 down-regulated (Additional file 3). Noticeably, a considerable proportion of DEGs corresponded to proteins with unknown function annotated as “hypothetical protein”. Among the upregulated DEGs, we found components of the interferon pathway (IRF8, 6.8x; IRF2, 3.0x and IFRD1, 2.8x), components of the Toll pathway (MyD88, 28.6x; IRAK4, 4.2x; cact, 3.4x; IKBKE, 2.3x) caspases (Casp-8, 2.8x and Casp-7, 2.0x), as well as other components known to be involved in oyster antiviral pathways (viperin, metalloproteinase inhibitors, baculoviral IAP repeat-containing proteins, dual specificity protein phosphatase 3, Bcl-2-like protein 1, SOCS2, Dual specificity mitogen-activated protein kinase kinase 3, Additional file 3). One gene, encoding for the interferon-stimulated enzyme ADAR-1, was strongly upregulated (6.4x) and its expression correlated with the OsHV-1 RNA levels in the six samples (Fig. 5b). Also, upregulated DEGs included several receptors possibly linked to the neuroendocrine system, like FMRF-amide, prostaglandin, dopamine, melatonin and IL-17 receptors as well as genes involved in the Pro-PO system (tyr-3 and Laccase-2, Additional file 3).

### Tracing the OsHV-1 transcription during infection

**Fig. 6:**
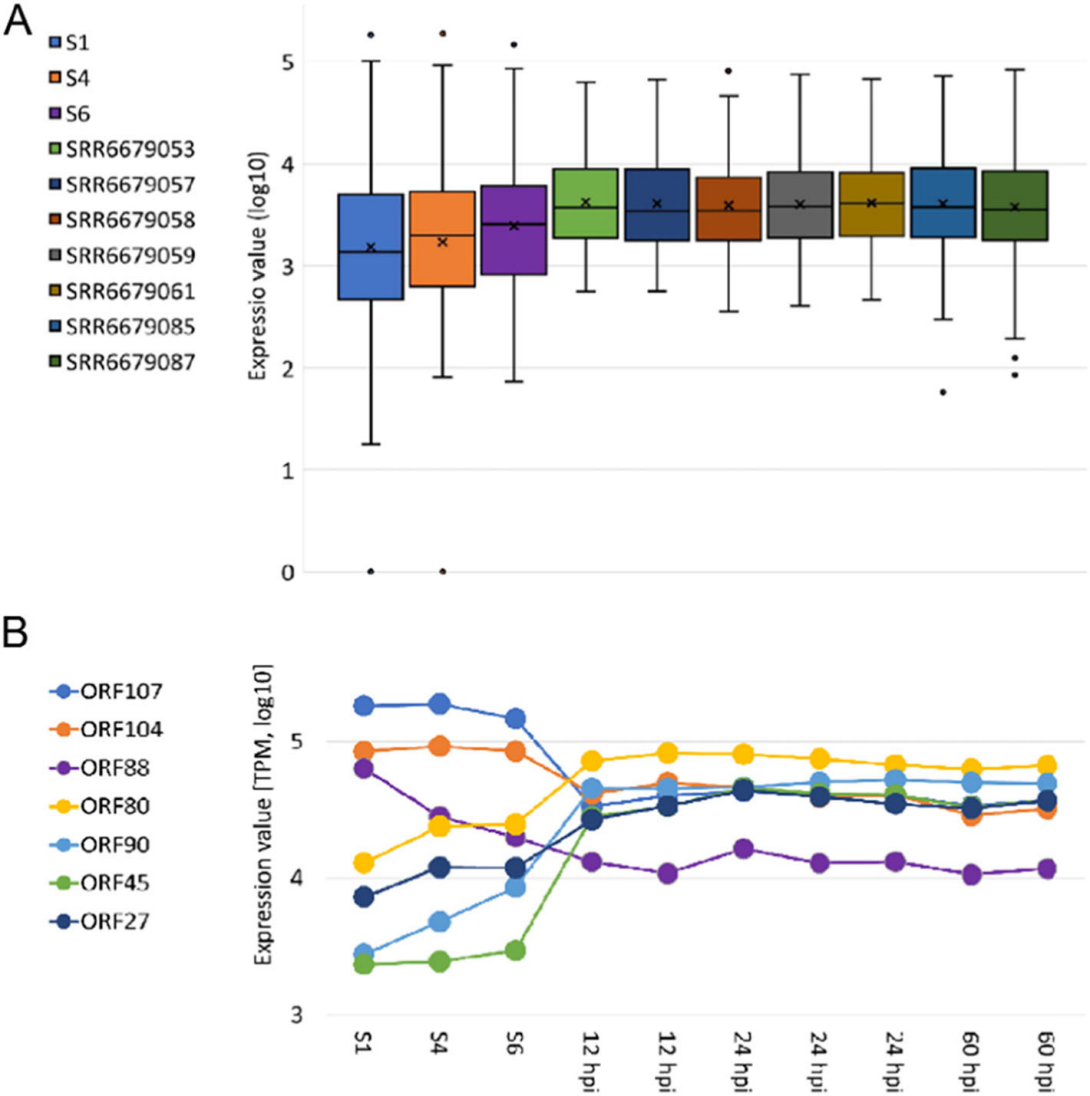
OsHV-1 expression analysis. **a.** The expression values of samples S1, S4 and S6 were compared with the ones obtained from 7 samples obtained from an experimental infection with OsHV-1 [18]. **b.** Expression profiles of selected OsHV-1 genes in all 10 samples (the samples of the experimental infection are named as time point (hpi, hours post infection)

We analyzed the OsHV-1 expression profiles in the 3 samples showing a sufficient number of viral reads (samples S1, S4 and S6, Table 1). We took advantage of the strand-specificity of our RNA-seq libraries to map the reads with directional constraints on the OsHV-1 genome, either in the expected coding-sense orientation, or as antisense mapping. While the “sense” mapping enabled us to get a more precise quantification of gene expression by counting the reads belonging to a given mRNA, the “antisense” mapping is a measure of antisense transcription levels, possibly produced by unknown viral transcripts located on opposite strands. The OsHV-1 “sense” profiles showed expression of most of the predicted genes, as previously reported for this viral family [18, 19, 22]. The most expressed OsHV-1 genes in our dataset were: ORF107, a protease called assemblin; ORF104 and ORF82, putative capsid proteins; ORF88, a possible membrane protein; ORF99, an apoptosis inhibitor. Other highly expressed viral genes denoted proteins with unknown functions, like ORF33, ORF119, ORF113, ORF41 and ORF127 (Additional file 4). Our samples showed lower averaged expression levels, but averaged expression peaks 2.4 times higher, in comparison to other seven RNA-seq samples resulting from experimental OsHV-1 infection (Fig. 6a). As a result of the heterogenous expression, the 5 most expressed OsHV-1 genes contributed 40–50% of the total expression in samples S1, S4 and S6, while they only contributed 21–26% of the total expression in previous studies [18, 19] (Fig. 6b). We also found that a small proportion of the reads from each sample (6.4–10.6%) mapped opposite to the expected coding directions. Most of these reads were however located at the ORF boundaries, suggesting that this phenomenon is caused by overlapping UTRs. This might reflect the dense distribution of OsHV-1 genes in combination with the lack of knowledge about the extension of OsHV-1 UTRs. In contrast, we also observed longer antisense signals along ORF100 (DNA polymerase) and ORF22, possibly indicating the presence of antisense genes that were not yet annotated (Additional file 5). Taking advantage of the abundant sncRNA reads, we aimed to investigate if OsHV-1 encoded genuine miRNAs. When grouping the putative OsHV-1 sncRNA reads into 19,997 clusters of identical reads, most clusters (97.3%) were represented by less than 10 reads. The distribution of OsHV-1 sncRNA read lengths was skewed towards shorter reads and did not show the distinctive peaks typical of sncRNAs (Fig. 2c-e). This distribution alone does not lend a lot of support for the presence of genuine miRNAs along the OsHV-1 genome. We nevertheless investigated the presence of possible structured RNAs further by using the VMir tool [46]. This resulted in the identification of 236 hairpins, covered by a total of 1456 sncRNA reads. However, the coverage graphs of these hairpins did not fulfill the minimal criteria for the identification of bona fide miRNAs. Therefore, based on our data, we suggested that OsHV-1 does not encode genuine miRNAs and that OsHV-1 sncRNA reads rather originated from mRNA degradation. Further evidence for a mRNA origin of the viral sncRNA reads comes from the matching SNP patterns in sncRNA and mRNA reads at positions edited by ADAR-1. The expression of oyster ADAR-1 correlated with the quantity of OsHV-1 RNA (Fig. 5b) and ADAR-1 exerts its enzymatic activity by editing dsRNA with a mechanism known as A-to-I editing, thus generating an identifiable footprint of ‘G’ mismatches, as we previously demonstrated [47]. In samples S1 and S6 we could identify 79 and 110 ADAR-mediated SNPs occurring with low frequencies (mean of 5.8 and 4%, respectively). Since almost all these edited positions could be traced also in the sncRNA reads, a degradation of full-length mRNA seems more likely than the expression of genuine viral-encoded miRNAs.

### miRNA/mRNA interactions during OsHV-1 infection

**Fig. 7:**
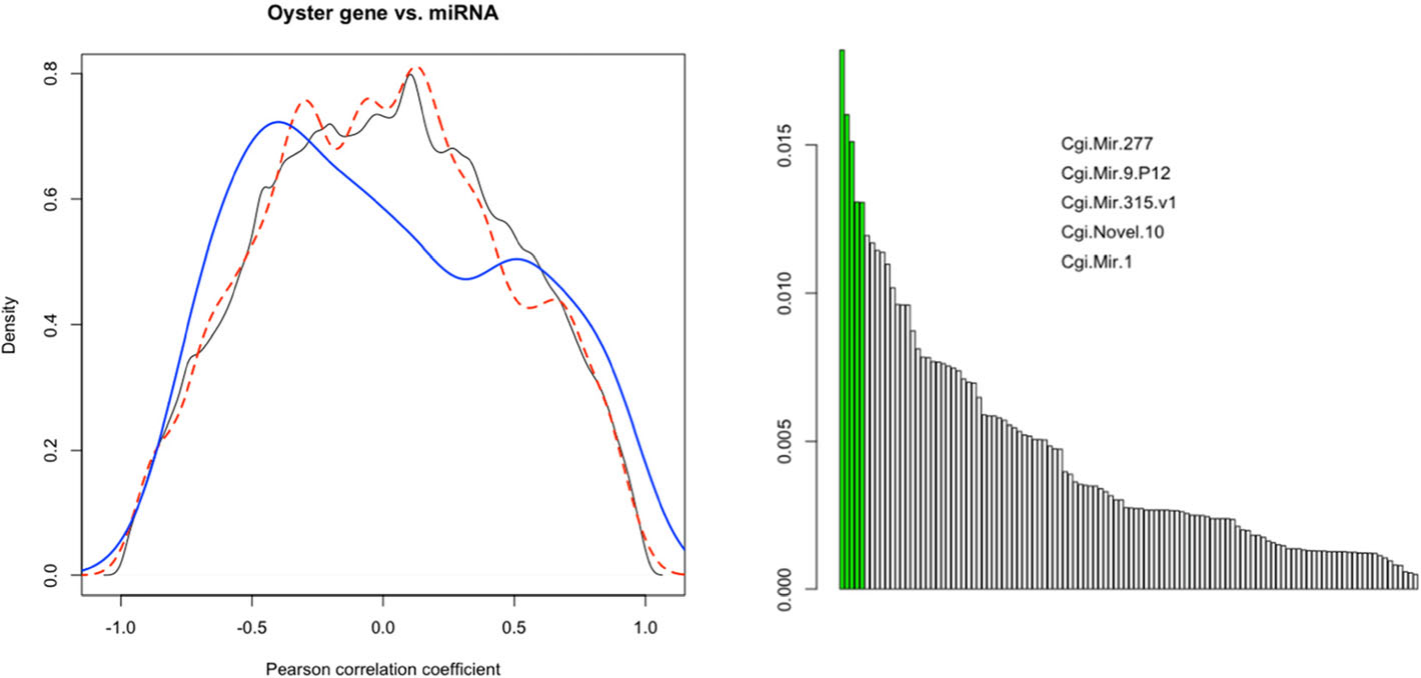
miRNA-mRNA expression correlation. **a.** Density plot of Pearson correlations for the combination of all oyster genes vs. all miRNA (black line), for all the miRNA-mRNA interactions predicted by *miranda* (red dotted line) and for the predicted interactions involving DE-miRNAs (blue line). **b.** Proportion of strong correlations for predicted miRNA-mRNA interactions (green bars correspond to the five miRNAs with the highest number of interactions)

Since miRNAs are expected to regulate the expression of coding genes by interacting with 3′-UTRs, we investigated the presence of possible miRNA-mRNA expression correlations among the six samples (Fig. 7). When comparing the correlations of predicted miRNA-mRNA interactions and especially the predicted interactions between differentially expressed DE-miRNAs and mRNAs with the Null distribution of all possible correlations we observed a lack of positive correlations. As expected, we found a comparatively strong increase of negative correlations, suggesting that miRNAs mostly repress gene expression [48]. In detail, the miRNAs with the highest proportions of strong correlations (i.e. the top and bottom 2.5% of all possible correlations) were *Cgi-mir-277, Cgi-mir-9, Cgi-mir-315, Cgi-mir-1 and Cgi-novel-10* (Fig. 6b). While *Cgi-mir-9* and *Cgi-mir-1* are highly expressed miRNAs with little variation among samples, *Cgi-mir-315* is included in the list of DE-miRNAs. Since UTRs are not annotated in the available oyster genome [49], we identified the 3′-UTRs by mapping our RNA-seq reads on the oyster genome with a mapper allowing the presence of large gaps due to introns. According to the available gene annotations, we could predict 2074 3′-UTRs longer than 30 nt. These sequences showed an average length of 441 nt. Using this dataset, *miranda* [50] predicted 1425 possible matches targeting 358 oyster genes. A total of 68 interactions, targeting 50 genes were assigned to the 15 DE-miRNAs (Table 3). However, only a few of these interactions affected differentially expressed oyster genes. The latter included Chloride channel protein 7, Laminin subunit beta-2, Collagen alpha-1(XIV) chain, Endo-1,6-beta-D-glucanase BGN16.3, Kelch-like protein 20, Achaete-scute-like protein 1, Tribbles-like protein 2 and two unknown proteins (Table 3). Next to the DE-miRNAs we also focused on miRNA-mRNA interactions of miRNAs that showed the highest proportions of strong miRNA-mRNA correlations (e.g. *Cgi-mir-277* and *Cgi-novel-10,* Fig. 6b), or showing the highest coefficient of variation among the six samples (*Cgi-mir-750* and *Cgi-mir-1175,* see Additional file 1). These two miRNA groups showed a similar number of matches, with few genes included in the DEG list. Among these, the match between *Cgi-mir-277* and a serine/threonine protein kinase stood out, that we found to be preferentially expressed in samples of OsHV-1-infected oysters.

**Table 3:** Putative miRNA-mRNA interactions. The possible miRNA-mRNA interactions are listed for the DE-miRNAs and for other miRNAs of interests. Bolded hits represent interactions involving DEGs. The gene IDs and putative annotations are reported according to the oyster genome annotation (v.9) and the IDs without annotation refer to proteins with unknown function (annotated as “hypothetical protein”). The underlined match to Histone deacetylase 6) refers to a miRNA-mRNA interaction described also in humans

miRNA ID	3′-UTR matches
**DE-miRNAs**	**Gene ID (functional annotation)**
*Cgi-Mir-10-P5*	CGI_10021820, **CGI_10022274 (Chloride channel protein 7)**, CGI_10022274, CGI_10022383 (Universal stress protein A-like protein), CGI_10026565 (Homeobox protein Hox-B7)
*Cgi-Mir-12*	CGI_10006175 (Lipoma HMGIC fusion partner-like 3 protein), CGI_10006175, CGI_10020896 (Vitamin D3 receptor A)
*Cgi-Mir-133-v1*	**CGI_10000616 (Laminin subunit beta-2)**, CGI_10015881 (Calcium-dependent protein kinase 31), CGI_10015881, CGI_10015881, **CGI_10016880 (Collagen alpha-1(XIV) chain)**
*Cgi-Mir-133-v2*	CGI_10001808 (26S protease regulatory subunit 7), CGI_10021971, CGI_10021971, CGI_10022309
*Cgi-Mir-193-P1*	CGI_10014060
*Cgi-Mir-1985*	CGI_10000475 (Aldose 1-epimerase), CGI_10016699 (Hexosaminidase domain-containing protein), CGI_10016780 (Spindle and kinetochore-associated protein 2), **CGI_10017457 (Endo-1,6-beta-D-glucanase BGN16.3)**, CGI_10017496 (Kinesin-associated protein 3), **CGI_10021840 (Kelch-like protein 20)**, CGI_10026811 (Inositol hexakisphosphate kinase 1), CGI_10027003 (Proteasome subunit beta type-3)
*Cgi-Mir-1993*	CGI_10021960 (Ras-related protein Rab-3), CGI_10021960, CGI_10021960
*Cgi-Mir-216-P1*	CGI_10017498 (Glycosyltransferase 25 family member 1), CGI_10017964 (Histone deacetylase 6), CGI_10020367 (Prostaglandin E2 receptor EP4 subtype), CGI_10021263 (Serine)
*Cgi-Mir-315-v1*	CGI_10000496, CGI_10016596 (Sacsin), CGI_10021927 (GTP-binding protein REM 1), CGI_10022452 (Small nuclear ribonucleoprotein E)
*Cgi-Mir-315-v2*	CGI_10000496, CGI_10009680 (Casein kinase II subunit alpha), CGI_10016245 (Rhophilin-2-B), CGI_10016506 (24-hydroxycholesterol 7-alpha-hydroxylase), CGI_10016596, CGI_10021971 (Myosin regulatory light chain sqh), CGI_10021971, CGI_10022309 (N-acetyltransferase 11)
*Cgi-Mir-375-P4*	**CGI_10009294**, CGI_10009754 (Histone-lysine N-methyltransferase, H3 lysine-9specific 3), CGI_10016585 (Calmodulin-like protein 11), CGI_10017336 (FERM domain-containing protein 1), **CGI_10017647 (Achaete-scute-like protein 1)**
*Cgi-Mir-92-o31*	**CGI_10001538 (Tribbles-like protein 2)**, CGI_10009355 (Cell division cycle 2-related protein kinase 7), CGI_10009355, CGI_10014759 (Guanine nucleotide-binding protein G(o) subunit alpha), CGI_10017574 (Protein still life, isoforms C)
*Cgi-Mir-92-o34*	CGI_10001538, CGI_10014759, CGI_10017574, CGI_10026245, CGI_10027167
*Cgi-Novel-19*	**CGI_10001640**, CGI_10015879 (Uncharacterized protein C21orf2), CGI_10022346 (Paxillin)
**other miRNAs**	
*Cgi-mir-277*	CGI_10013946 (Serine), **CGI_10014307 (Serine/threonine-protein kinase CTR1)**, CGI_10020584
*Cgi-novel-10*	CGI_10013878 (ADP-dependent glucokinase), CGI_10020119 (MAP kinase kinase win1), CGI_10020428 (Latrophilin-3), CGI_10020556 (RAD50-interacting protein 1), **CGI_10021961**, CGI_10026543 (Membrane progestin receptor gamma-B), **CGI_10026562 (Homeobox protein LOX2)**
*Cgi-mir-750*	CGI_10014483 (Contactin), **CGI_10016707**, **CGI_10017867**
*Cgi-mir-1175*	CGI_10001636 (Cytochrome P450 2D28), CGI_10014558 (Heat shock 70 kDa protein 12A), CGI_10014838 (Thrombospondin type-1 domain-containing protein 4), CGI_10017449 (Transcription factor HES-1-B), CGI_10027096 (Activin receptor type-1)

Using *miranda* we also identified 307 matches between oyster miRNAs and the OsHV-1 genome, and 24 of these involved DE-miRNAs. However, the absence of information regarding the extent of viral UTRs as well as the low number of samples with enough mRNA data (*n* = 3) prevented the possibility to link miRNA matches to a given viral gene.

## Discussion

To increase our understanding of the mechanistic role of sncRNAs as gene expression regulators during OsHV-1 infection in oysters, we simultaneously sequenced the sncRNAs and mRNAs from six oysters naturally infected with OsHV-1 in the Goro lagoon (Italy). While we could clearly see the onset of antiviral oyster immunity in response to OsHV-1 infection, expression patterns of oyster miRNAs differed from mRNA transcription in several aspects. Most obvious was the clustering of miRNAs according to virus activity, which was set off against a grouping by viral transcript abundance. Furthermore, only few of the miRNA-mRNA correlations affected antiviral genes responding to OsHV-1 infection. This lack of consistency between miRNA and mRNA profiles related to the antiviral response might be a sign of the inherent variability occurring in natural, uncontrolled, infections. Alternatively, the weak correlation between miRNA and mRNA transcription profiles may indicate a limited regulatory role of miRNAs in oyster antiviral processes. Either way, our exploration of the miRNAome landscape in response to OsHV-1 infection indicated sophisticated miRNA regulatory networks with only loose connections to the oysters’ antiviral immune response.

### Expression of miRNA diversity in oysters

By using only the 151 miRNA predictions available for oyster in the MirGeneDB [44], we adopted a conservative approach that can prevent the inclusion of false positive miRNAs, typically found in sncRNA sequencing studies of model organisms [45, 48, 51]. Expression analysis revealed that Cgi-mir-10-P2 (previously reported as mir-100), Cgi-mir-279, Cgi-mir-184-P7 and Cgi-mir-1 accounted for most of the sncRNA reads, making Cgi-mir-184-P7 the most suitable housekeeping miRNA for data normalization. The high expression levels of these miRNAs is in agreement with previous studies in oyster [38, 52], *Chlamys farreri* [43] and *Tegillarca granosa* [53]. For other miRNAs (like *Cgi-mir-67* or *Cgi-let-7*), we measured lower expression levels than those reported before [38, 52]. We also observed reversed amounts of mapped reads between the mature and star arms for some miRNAs (*Cgi-mir-36, Cgi-mir-1992, Cgi-novel-1, 2, 8, 13* and *Cgi-novel-15*). Although both arms can be selected to produce mature and functional miRNAs, a preference towards one of the two arms is the rule [26], resulting in a bias of the number of mapped reads among arms. The mechanism allowing such a selection is still unknown and arm-imbalance was recently reported as a way to modify miRNA targets during cancer [54]. However, most of the observed “reversions” came from novel oyster miRNAs, possibly because limited knowledge is available on these new oyster-specific miRNA families.

Overall, we identified 15 oyster miRNAs that were differentially expressed among samples. The resulting miRNA expression profiles suggested a sample grouping according to the ratio of OsHV-1 RNA over DNA (i.e. δ values, the relative transcriptional activity), whereas expression of *C. gigas* coding genes rather grouped according to absolute viral transcription levels. This would indicate the existence of more subtle regulative mechanisms depending upon OsHV-1 stage that control miRNA expression, compared to the regulation of coding genes. Notably, some of the DE-miRNAs were located in clusters in the oyster genome (*Cgi-mir-750* with *Cgi-mir-1175* and *Cgi-mir-12* with *Cgi-mir-216*) and showed similar expression values between δ groups, suggesting co-expression and potential synergistic functions, previously shown for humans [55]. Several of the DE-miRNAs belong to miRNA families that were previously described to modulate immunity during viral infections in other organisms. This included *mir-12* that was found upregulated in WSSV-infected shrimps, where it modulates phagocytosis, apoptosis and antiviral immunity [56]. Two other miRNAs, *mir-375* and *mir-750*, were also highly responsive to WSSV infection in *Panaeus monodon* [57]. Additionally, *mir-315* regulated the pro-PO system during WSSV infection, thereby inhibiting the spread of the virus [58]. Both in sncRNA-seq and RT-qPCR data, *Cgi-mir-750* was one of the most induced miRNAs in samples with high OsHV-1 infection and it was the miRNA with the highest number of matched among DEGs.

**Fig. 8:**
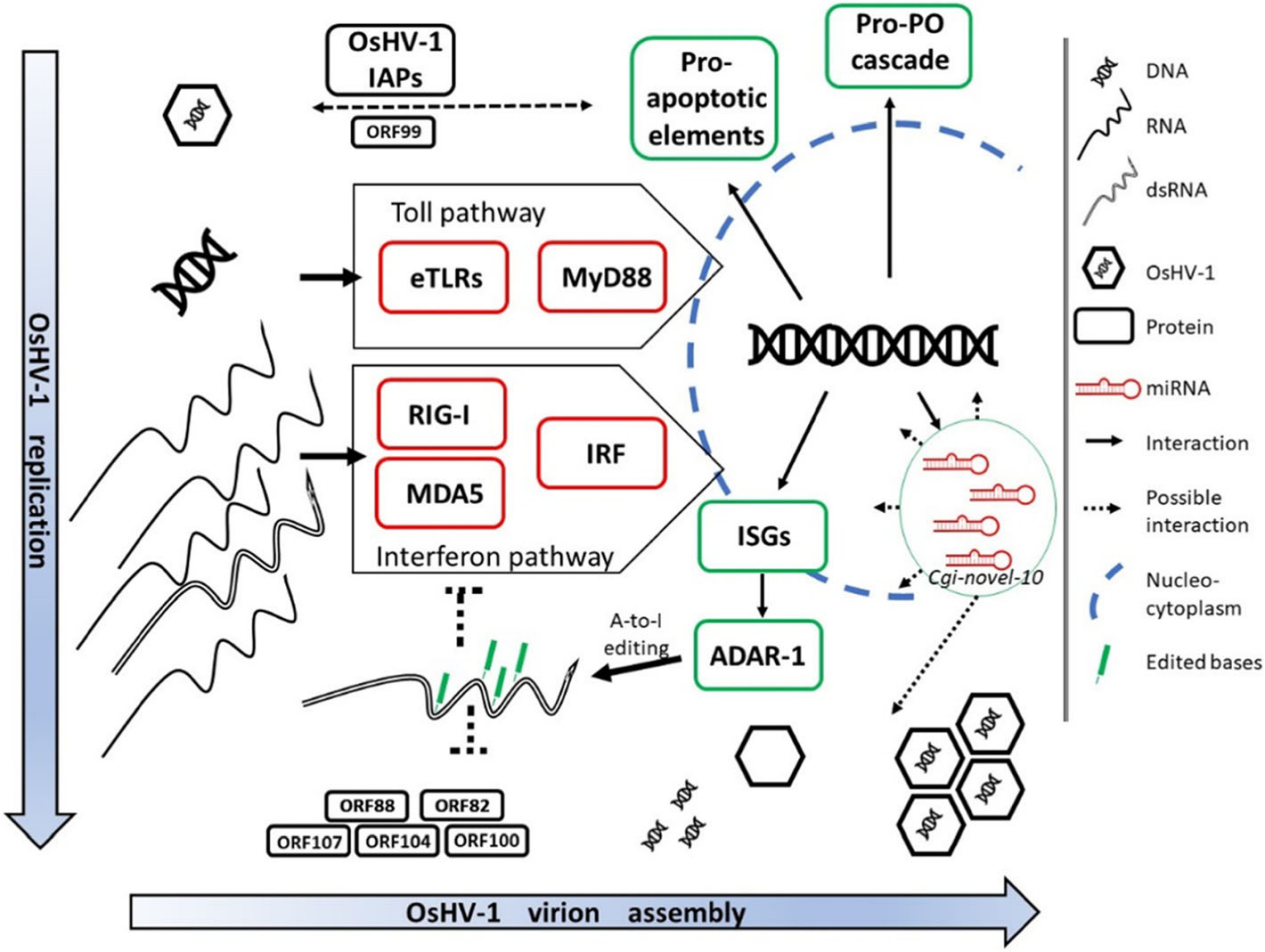
Conceptual model of possible molecular events describing OsHV-1 and *Crassostrea gigas* interactions in the infected oyster cells. Replicating OsHV-1 DNA amplifies the production of viral RNAs, dsRNAs and proteins necessary for virion assembly and responsible of some host-pathogen interactions (e.g. OsHV-1 IAPs). The binding of viral DNAs and dsRNAs to specific oyster receptors, namely endogenous TLRs (eTLRs) and RIG-I/MDA5 proteins, activates the Toll and Interferon pathway, respectively (red boxes) and leads to the transcription of antiviral effectors (green boxes). Pro-apoptotic genes, like caspases, Pro-PO elements, like tyrosinases and laccases, and interferon stimulated genes, like viperin and ADAR-1 are upregulated during OsHV-1 infection. These antiviral effectors control the virus, which counteracts by expressing anti-apoptotic viral genes (IAPs, like ORF99). Oyster ADAR-1 edits dsRNAs with a mechanism known as A-to-I editing, producing G mismatches that impair dsRNAs, and possibly making the edited dsRNAs less effective in activating dsRNA receptors, while the impact on OsHV-1 replication is unknown. A few oyster miRNAs are regulated during OsHV-1 infection, but their function in controlling host and viral genes remain unclear

As previously reported by using SSH libraries [59], an upregulation of pro-PO related genes could be detected during OsHV-1 infection and an increase of the PO activity was also evident in *C. farreri* infected with a OsHV-1 congener [60]. Accordingly, we found a few pro-PO related DEGs upregulated in samples showing OsHV-1 activity, including a laccase and a tyrosinase. A similar laccase (lac-2) was recently reported as strongly upregulated during WSSV infection in *Litopenaeus vannamei* [61]. Among the oyster’s countermeasures against the OsHV-1 infection we can include the interferon-like pathway, the toll-pathway as well as apoptosis and pro-PO activity in a conceptual model of the oyster response to OsHV-1 infection (Fig. 8). Moreover, the trace of ADAR-1-mediated editing of viral dsRNAs in samples S1 and S6, together with a good correlation between ADAR-1 expression and OsHV-1 transcription confirm our previous findings suggesting the main role of this enzyme in editing exogenous dsRNAs [47], although the biological meaning of this editing during oyster-OsHV-1 interaction is still unknown.

Conceivably, all these well-known antiviral pathways are poorly interconnected with the predicted miRNA matches. In fact, for some predicted miRNA-mRNA interactions we found strong correlations in expression values indicative of miRNA-mediated co-regulation processes. However, we could only identify a limited number of possible interactions between miRNAs and 3′-UTRs of mRNAs. As regards the matches of DE-miRNAs reacting to OsHV-1 infection, we found that these matches mostly involved genes that were neither directly related to antiviral responses, nor included in the list of DEGs. The possible interaction between *Cgi-mir-277* and a serine/threonine protein kinase likely represents an exception here, since this transcript was upregulated in our and other OsHV-1-infected samples, and serine/threonine protein kinases are known to be involved in differential resistance to viral infection [62]. Therefore, *cgi-mir-277* could target a functionally conserved mechanism in viral immune responses. The other DE-miRNAs may be involved in indirect regulative mechanisms or target gene transcripts not yet known to be involved in antiviral oyster processes, but overall, the regulatory role of miRNAs in the antiviral response of the oyster seems to be limited.

### Interaction of miRNAs with viral genes

Oyster miRNAs could also target viral infections by binding to viral genes and thereby limiting or promoting viral gene expression. Out of the prediction of 307 positions the OsHV-1 genome, 40 matches were exclusive to oyster miRNA families, which might be a result of co-evolution between *C. gigas* and OsHV-1. And while we found several possible miRNA-OsHV-1 matches including highly expressed viral genes (e.g. ORF88) or genes in the vicinity of highly expressed genes (e.g. ORF108 near ORF107, i.e. the most expressed OsHV-1 gene), we could not find compelling evidence for a functional relationship between oyster miRNAs and viral genes due to the low number of samples with sufficient viral reads. Furthermore, the extent of OsHV-1 UTRs is presently unknown and the high rate of antisense transcription that we found is a clear indicator for the poorly understood complexity of the OsHV-1 transcriptome. Based on this and on the fact that most of the OsHV-1 genes have unknown function, any assumption of a functional meaning of these matches would be very speculative. Yet, the enrichment of negative correlations in predicted miRNA-mRNA interactions along with the previously reported matches from other organisms still suggests, that the miRNA machinery can play an important role in regulating transcription in oysters, but this role might be limited for the interaction with OsHV-1.

For the virus itself, it was not yet clear whether it utilizes miRNA to regulate its own transcription or to modulate host immunity. Our search for OsHV-1-encoded miRNAs suggested that OsHV-1 does not utilize miRNAs. This was not self-evident since other viruses related to OsHV-1 [6], as well as WSSV [16, 63] can encode miRNAs. However, the OsHV-1 sncRNA reads showed a size distribution lacking abundant size classes typical of miRNAs. While genome-wide prediction tools suggested the presence of RNA hairpins along the OsHV-1 genome [16], our data did not support evidence for increased expression of these loci, nor coverage profiles consistent with the presence of genuine miRNAs. Therefore, we rather suggest that OsHV-1 sncRNA reads originated from degraded mRNAs. Also, the size profile of OsHV-1 sncRNA reads suggested that RNAi is not active against OsHV-1 in oyster, since a size-bias would be expected in case of RNAi-mediated RNA degradation [64]. RNAi was reported during WSSV infection in shrimp [35] as well as in other arthropod species [65], but this study rather supports previous findings indicative of a marginal role for RNA interference in the antiviral response of bivalve species [66, 67].

## Conclusions

The parallel characterization of sncRNA and mRNA data from six oysters naturally infected with OsHV-1 revealed the revealed the regulatory network of the oyster miRNActome. Generally, miRNAs could serve molecular targets for future studies trying to control disease outbreaks. While the overall miRNA expression profiles and some specific miRNAs (for example, *Cgi-mir-750, Cgi-mir-277, Cgi-mir-375*) correlated with the transcriptional activity of the virus, we found only few predicted interactions with genes known to be involved in viral immunity. In contrast, we observed large differences in the mRNA transcriptional profiles, characterized by the upregulation of several antiviral and immune pathways. The miRNAs transcription profiles, however, showed differential expression of few miRNAs possibly linked to their regulative roles in the onset or in the control of the OsHV-1 infection. The coupling of mRNA and sncRNA sequencing showed that while several major immune pathways are activated during an infection, with miRNAs only playing a limited direct role in the viral infection of oysters (Fig. 8). Nevertheless, the altered expression profiles of miRNAs in response to viral transcriptional activity could indicate that more indirect roles exist for some of these miRNAs possibly leading to functional consequences in terms of host-pathogen interactions (e.g. Cgi-novel-10, Fig. 8).

## Methods

### *C. gigas* deployment in open field and sampling

Triploid *Crassostrea gigas* seed of French origin (T6 size, 0.15 g on average) was placed in lantern-like baskets at 0.5–1 m depth in the Goro lagoon (44°48.728′N 12°17.905′E, North Adriatic Sea, Italy) on March 19th, 2016. The growing oysters were sampled from Apr 26th to Jun 21st (26/4, 03–10–17-24-31/05 and 07–14-21/06), a time span which previously included OsHV-1 infections and mortality [68]. Within a month the oyster spat reached a shell length of 2.46 ± 0.12 cm (*N* = 30, 17th May) and continued to grow during the whole monitoring period (Additional file 6). From the oysters collected on May 17th (2.3–2.7 cm shell length, *N* = 45), a fragment of mantle and the whole gills were individually dissected on ice, immerged in RNA Stabilization Reagent (RNAlater, Qiagen, Milano, Italy) and stored at − 80 °C. Fragments of mantle and gill tissues were used for OsHV-1 DNA quantification and for individual RNA purification.

### Analysis of OsHV-1 DNA

The presence of OsHV-1 DNA was measured by quantitative PCR. Total DNA was extracted from ~ 25 mg w.w. of oyster gill and mantle using the QIAamp® DNA Mini Kit following the manufacturer’s instructions (Qiagen). The purified DNA samples were quantified with a NanoDrop spectrophotometer (ThermoFisher Scientific, Waltham, MA, USA) and diluted to 5 ng/μl for the amplification reaction targeting the catalytic subunit of the viral DNA polymerase (AY509253 ORF100) [69]. Five μl of DNA were added to a qRT-PCR reaction mix composed of 12.5 μl SsoFast™ EvaGreenR Supermix (Bio-Rad Laboratories, Segrate, Milano), 1.25 μl of each primer (HVDP-F: 5′ ATTGATGATGTGGATAATCTGTG 3′; HVDP-R: 5′ GGTAAATACCATTGGTCTTGTTCC 3′) diluted at the concentration of 0.5 μM, and 5 μl of water. The amplification reactions were performed in a Rotor-Gene Q thermocycler (Qiagen) as follows: 1 cycle of polymerase activation at 95 °C for 5 min; 40 cycles of amplification at 95 °C for 30 s, 60 °C for 1 min, and 72 °C for 45 s and a final step for melting temperature curve analysis from 65 to 95 °C (10 s/step, ramp rate 0.5 °C/sec) [70]. The absolute number of OsHV-1 DNA copies/μl was determined by comparing the C_t_ values resulting from a standard curve. Plasmidic DNA including the OsHV-1 target region was serially diluted 1:10 in the range 10–10^6^ DNA copies/μl and used to compose the standard curve.

### RNA extraction, library preparation and sequencing

Total RNA was extracted from individual oyster gills according to the Trizol manufacturer’s instructions (Thermofisher Scientific), quantified with a Qubit 2.0 fluorometer (Thermofisher Scientific), and the RNA quality was tested with an Agilent bioanalyzer using the RNA6000 pico kit by automated electrophoresis and fluorescence signal detection (Thermofisher Scientific). A Real Time PCR (qRT-PCR) approach was used to estimate the OsHV-1 transcription levels in individual oysters. Briefly, the expression values of OsHV-1 ORF104 and oyster housekeeping gene *Elongation factor 1-alpha* (El1α) were compared using the delta Ct method [71]. One μg of total RNA per sample was used to prepare sncRNA and mRNA libraries. In particular, sncRNA libraries were prepared according to TruSeq Small RNA Library Prep Kit (Illumina), whereas mRNA libraries were based on poly(A) RNA-selection or rRNA-depletion. Both mRNA library types were prepared according to the Illumina paired-end technology to retain the strand information of the reads (stranded libraries). The poly(A) library was prepared using the mRNA-Seq library Prep Kit V2 (Lexogen, Austria) following the manufacturer instructions, while the Ribo-0 library was prepared according to the TruSeq Stranded Total RNA kit (using a Ribo-zero Gold kit, Illumina, US). HT-sequencing was outsourced and carried out on a HiSeq 2500 instrument (2 × 150, Admera Health, USA) or on a HiSeq High-Output v4 instrument (2 × 125, DNA Sequencing Center at Brigham Young University, USA).

### Analysis of small non-coding RNA-seq data

High-throughput sncRNA data were trimmed using cutadapt v.1.18 [72] implemented into Trimgalore! v.0.6.0 (https://www.bioinformatics.babraham.ac.uk/projects/trim_galore/), setting the minimal quality threshold to PHRED25 after removing adaptor sequences. FastQC (https://www.bioinformatics.babraham.ac.uk/projects/fastqc/) was used to verify the absence of sequencing adaptors as well as to verify the quality statistics of each dataset. The clean reads were uploaded in CLC Genomic Workbench v.12.0 (Qiagen, US), they were size selected (18–40 nt) and mapped on the oyster and OsHV-1 reference genomes (GCA_000297895 [49] and MG561751 (OsHV-1) [73], respectively) as well as on the predicted oyster miRNA precursors obtained from MirGeneDB (http://mirgenedb.org/) [44], applying 0.9 and 1 for similarity and length fraction parameters, respectively. The mapping graphs of the 151 oyster miRNA precursors were manually inspected to verify the presence of the minimal annotation criteria. These included the presence of coverage for both miRNA arms, absence of reads mapped in the surroundings of the annotated miRNAs and 5′ read homogeneity. Subsequently, the sncRNA reads were clustered per sample and only perfect matches and were counted. Only clusters with a minimal representation of 100 reads were further considered and annotated according to the MirGeneDB oyster entries. miRNA expression values were calculated per sample as number of mapped read and compared using a proportions-based test [74] with FDR-corrected *p*-values. miRNAs with p-value lower than 0.01 and absolute fold-change higher than 2 were considered as differentially expressed (DE-miRNAs).

### Annotation of oyster UTRs and miRNA/mRNA expression analysis

To identify the 3′ untranslated regions (3′-UTRs) *of C. gigas* mRNAs, we mapped the mRNA reads on the oyster genome (oyster_v9) using the large gap mapper implemented in the CLC, that allowed the presence of gaps due to introns. The resulting mapping was used to extend the predicted genes (limited to CDS regions) to untranslated regions. 3′-UTRs longer than 30 nt were counted and used, together with the OsHV-1 genome, to verify the presence of putative miRNA targets using *miranda* [50], applying a conservative minimal score of 155 and a minimal energy of − 20. To investigate the interaction of miRNAs and mRNA we computed Pearson’s correlation coefficient for all possible combination of the expression values of all genes and all miRNAs with expression values to obtain a Null distribution of all possible correlations. We then took subsets of a) all the interactions predicted by miranda and b) the interactions involving only differentially expressed miRNAs among these predicted interactions. The distributions of both predicted sets were then evaluated against the null distributions to identify regions where predicted interactions deviated from the null distribution.

### Selection of the best OsHV-1 reference genome

Trimgalore was used to trim RNA-seq reads, applying a minimal quality of PHRED30, a minimal read length of 80 bp, and only validated paired reads were considered. To determine the bivalve *Malacoherpesviridae* most suitable as a reference genome, the whole read dataset was mapped on three available OsHV-1-μvar genomes, applying 0.95 and 0.95 of similarity and length fraction parameters, respectively. Read mapping resulted in similar matches to the three genomes, while SNP calling identified 59, 83 and 80 variants, for MG561751 (Italy), KY242785 and KY271630 (France and Ireland) genomes, respectively. Based on few synonymous and non-synonymous SNPs and considering the geographical vicinity of the OsHV-1-PT isolate (Porto Tolle, North Adriatic Sea), we selected MG561751 genome as reference for the purposes of this study.

### Analysis of poly(a) tail selection and rRNA ribosomal RNA depletion performances

Preliminary to proceed with high-throughput sequencing, we tested the performance of an rRNA depletion approach in comparison with poly(A)-tail selection on one sample (S6). To calculate the amount of *C. gigas* and OsHV-1 reads (defined as on-target reads), all clean reads were mapped to the oyster and OsHV-1 reference genomes using the CLC *mapping* tool (Qiagen, Denmark), setting 0.8 and 0.5 for the similarity and length parameters, respectively. Unmapped reads were collected and remapped on the reference genomes while allowing the presence of large gaps (introns or large structural variations), using the CLC *large gap read mapping* tool, and applying 0.9 and 0.9 for the similarity and length parameters, respectively. The remaining unmapped reads were *de-novo* assembled using the CLC *assembler* tool (with a minimal contig length of 200 bp, bubble and word sizes set to automatic) and the obtained contigs were subjected to ORF prediction with the *transdecoder* tool by applying default parameters [75]. According to ORF predictions, the contigs were preliminary classified into coding or putative non-coding transcripts and were blasted (*blastx*) against the NCBI nr-protein database (downloaded the 10th of Sept. 2018). Blast results were used for species assignment, sequence annotation and identification of possible lncRNAs using the Blast2GO suite [76]. The identification of conserved domains on the predicted proteins was carried out with HMMer v.3.1 based on the whole Pfam-A domain collection (cut-off E-value of 10^− 5^) [77]. Putative non-coding transcripts were further screened for the presence of conserved RNA structures, using the Rfam v.13.0 database [78] with Infernal v1.1 [79].

To estimate the level of rRNA depletion, the trimmed reads of poly(A) and Ribo-0 libraries were mapped on a reference oyster rRNA sequence, obtained concatenating the 13 rRNA sequences annotated in the *C. gigas* genome as well as other oyster rRNAs annotated as ‘hypothetical protein’, identified by *blastn* against the nr NCBI database. The level of strand specificity of the libraries was determined by mapping the trimmed reads of each library on 7 oyster housekeeping genes [80], selected for the absence of known antisense transcription (EKC19952, *Ubiquitin-conjugating enzyme E2D2*; EKC42233, *S-phase kinase-associated protein 1*; EKC41722, *Heterogeneous nuclear ribonucleoprotein A2/B1*; EKC37135, *Heterogeneous nuclear ribonucleoprotein Q*; EKC32788, *Eukaryotic translation elongation factor 2*; EKC23295, *Glyceraldehyde-3 phosphate-dehydrogenase* and EKC33063, *Elongation factor-1α*). The coverage graphs were manually inspected to exclude the possible presence of non-annotated antisense transcripts. Subsequently, the number of reads mapped in the sense direction over the total number of mapped reads was taken as an estimation of the strand-specificity of the library, according to [81]. Limited to viral reads, and to better compare viral expression profiles between the two libraries and between sense and antisense directions, the expression values were computed both as total number of mapped reads and as Reads Per Kilobase Million (RPKM) either mapping the reads on the whole genome or, separately, on each viral ORF. In line with other reports [82], we showed that poly(A) selection underestimates the expression levels of non-polyadenylated transcripts like ncRNAs and histones, but, on the contrary, it produces a higher amount of on-target reads. This latter point was the reason for using poly(A) selection for this study. However, we still considered ribosomal rRNA depletion as a valid alternative for future investigations, probably useful to reveal OsHV-1 non-polyadenylated transcription not easily detectable with poly(A) reads. Further details are reported in Additional file 7.

### RNA-seq expression analysis

Expression profiles were computed by mapping all clean reads on the virus and host genomes, applying 0.8 for both the length and similarity parameters. Owing to the stranded libraries, reads were mapped using a strand constraint (either sense or antisense mapping). For *C. gigas*, the expression values were computed as Transcript Per Million (TPM) to normalize for the different sequencing yield [83], whereas for OsHV-1 we used Reads Per Kilobase Million (RPKM) values because of the high difference in the number of total mapped reads among samples. Genes were regarded as differentially expressed (DEG) if presenting an absolute fold change higher than 2 with an FDR corrected *p*-value lower than 0.01 (Baggerley’s test).

### Validation of miRNA expression by RT-qPCR

The expression levels of 8 selected miRNAs were tested by RT-qPCR using the miRCURY LNA miRNA SYBR Green PCR kit (Qiagen). The LNA primers were purchased from catalogue products, if available, or designed using the GeneGlobe platform (https://geneglobe.qiagen.com/) (Additional file 2). First-strand cDNAs were synthesized by starting from 50 ng of total RNA of samples S1-S8 and using the miRCURY LNA RT Kit (Qiagen), according to manufacturer’s instructions with the following cycle: 60 min at 42 °C, for 5 min at 95 °C and immediately cooled down to 4 °C. The obtained cDNAs were mixed in a unique pool and 5 dilutions were prepared, from 1:10 to 1:200 to test primer efficiency in a preliminary RT-qPCR plate. All the designed primers showed a high efficiency (r^2^ > 0.95) when tested over serial cDNA dilutions, with the single exception of *Cgi-Novel-19* primer (0.88 of efficiency). Final RT-qPCR reactions were carried out using 3 μl of 1:100 cDNAs in a 10 μl of final reaction mixture (5 μl of 2X Master Mix, 0.5 μl of Rox passive reference dye, 1 μl of primer, 0.5 μl of water). Amplification cycles were performed on an Applied Biosystems 7900HT Fast Real-Time PCR System in a MicroAmp Fast Optical 384-Well Reaction Plate (Life Technologies) as follows: 95 °C for 2 min and 40 cycles of 95 °C for 10 s and 56 °C for 1 min. At the end of the reaction, a dissociation curve analysis was performed to ascertain the primer specificity. Each qPCR assay was carried out in triplicate on the same plate for each primer. Two stable housekeeping miRNAs were chosen as reference (*Cgi-mir-10-P2* and *Cgi-mir-184-P7*). The relative expression ratio of the selected target gene was based on the delta–delta Ct method (2 − ΔΔCt) [71].

## Supplementary information

**Additional file 1.** miRNA expression values. miRNA ID according to MirGeneDB, average expression value, percentage of total miRNA expression, expression levels in the six samples and coefficient of variation of the expression values are reported for 132 oyster miRNAs. miRNAs reported in bold resulted differentially expressed (Table 2), while underlined miRNAs contributed less to 0.01% to the total expression.

**Additional file 2.** RT-qPCR analysis of selected miRNAs.

**Additional file 3 **mRNA expression values. Gene ID, fold change, FDR-corrected *p*-value, gene description and the six expression values as TPM are reported for the 403 differentially expressed genes in the comparison between samples S1, S4 and S6 versus S2, S3 and S5. Gene directly involved in oyster antiviral pathway are highlighted in yellow, whereas other genes discussed in the text are highlighted in light blue.

**Additional file 4.** OsHV-1 expression values. Gene ID, expression values in samples S1, S4, S6 plus in other 7 datasets are reported for the OsHV-1 genes.

**Additional file 5.** RNA-seq coverage of the OsHV-1 genome obtained with Ribo-depleted and polyA-selected libraries generated from sample S6. The coverage graph along the 204 kb OsHV-1 genome was reported in a 0-3000x scale for the Ribo-0 library mapped in sense direction (A), in antisense direction (C) and for the poly(A) library in sense direction (D) and antisense direction (F). The red arrows (B and E) depicted the OsHV-1 ORF annotations. The blue rectangle highlighted the viral DNA polymerase, ORF100.

**Additional file 6.** Oyster sampling data.

**Additional file 7 **Comparison of two mRNA enrichment methods for dual RNA-seq analysis of *C. gigas* infected with OsHV-1.

## Data Availability

The raw reads are deposited in the NCBI Short Reads Archive under project accession IDs PRJNA484109. For comparative purposes, other RNA-seq datasets were retrieved from the NCBI SRA database (PRJNA423079).

## Abbreviations

cDNA: complimentary DNA
CDS: Coding sequence
DE: Differentially expressed
HaHV-1: Haliotid herpesvirus-1
HTS: High-throughput sequencing
miRNA: microRNA
mRNA: messenger RNA
ncRNA: non-coding RNA
OsHV-1: Ostreid herpesvirus-1
RPKM: Reads Per Kilobase Million
RT-qPCR: Quantitative reverse transcription PCR
sncRNA: short non-coding RNA
TPM: Transcript Per Million
UTR: Untranslated region
