# Supplementary material for: Parallel analysis of miRNAs and mRNAs suggests distinct regulatory networks in Crassostrea gigas infected by Ostreid herpesvirus 1
Source: BMC Genomics. 2020 Sep 10;21:620. doi: 10.1186/s12864-020-07026-7 (PMC7488030; doi:10.1186/s12864-020-07026-7)
Supplement: Supplementary file 2 — Additional file 2. RT-qPCR analysis of selected miRNAs. [file 12864_2020_7026_MOESM2_ESM.pdf]

Additional File 2. RT-qPCR analysis of selected miRNAs.

S. File 2, Table 1. Primer sequences. For 8 seleceted miRNAs (2 possible normalizer, 4 DE-miRNAs and 2 additiona miRNAs) are reported the miRNA name, their classification, size, sequence and the MIMAT code identificative for the designed primer (GeneGlobe platform, Qiagen.com).

Name	Note	Size	Start of sequence	MIMAT code
Cgi-Mir-133	DE-miRNA	22	UUGGUCCCCUUCAACCAGCUGU	MIMAT0000340
Cgi-Mir-315	DE-miRNA	23	UUUUGAUUGUUGCUCAGAAAGCC	MIMAT0008944
Cgi-Mir-1985	DE-miRNA	23	UGCCAUUUUUAUCAGUCACUGUG	MIMAT0009716
Cgi-Novel-19	DE-miRNA	23	AGCUAUAAUGGUUGUCAUUUGUA	**YCP0054702**
Cgi-novel-10	not DE	22	GAUACGACUAGGUUGUACGUGU	**YCP0054708**
Cgi-mir-750	not DE	23	CCAGAUCUAACUCUUCCAGCUCA	MIMAT0009547
Cgi-Mir-10	normalizer	22	AACCCGUAGAUCCGAACUUGUG	MIMAT0000098
Cgi-Mir-184-P7	normalizer	22	UGGACGGAGAACUGAUAAGGGC	MIMAT0000331

S. File 2 Figure 1. Correlation between sncRNA-seq and RT-qPCR data. For the 6 target miRNAs, a dispersion plot was used to visualize the correlation between expression levels measured by HT-seq and RT-qPCR. Data were normalized using Cgi-Mir-184-P7 as reference.

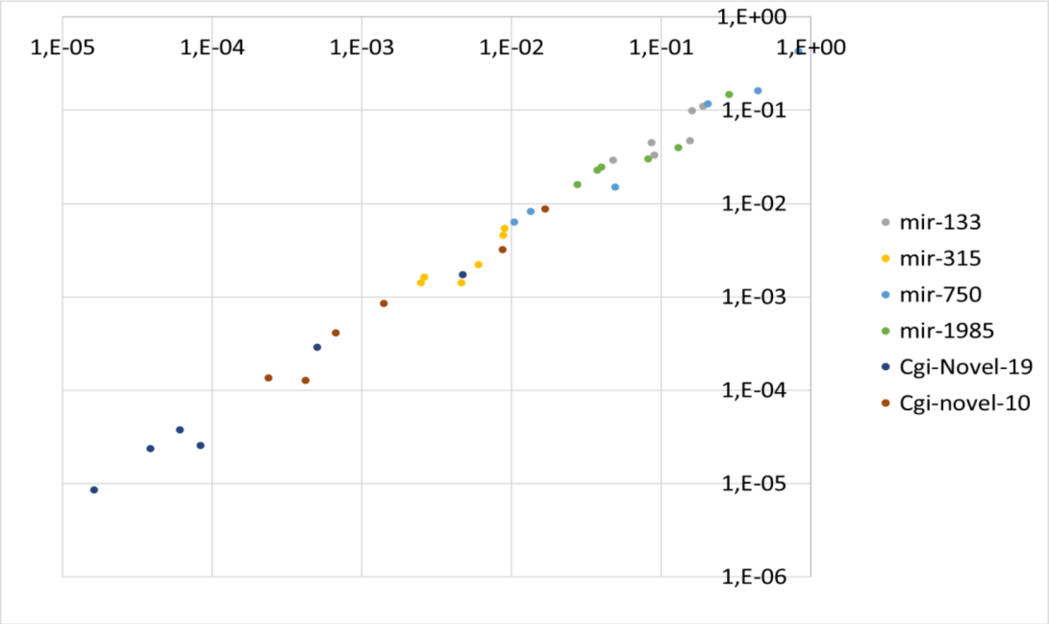

S. File 2, Figure 2. Fold changes computed using sample S5 as control.

FC vs. S5	S1	S2	S3	S4	S6	S7	S8
**mir-133**	0,25	0,85	0,47	0,82	0,45	0,38	0,64
**mir-315**	3,63	1,06	2,45	1,87	3,57	2,01	6,09
**mir-750**	0,05	0,07	2,17	0,24	4,03	0,65	3,87
**mir-1985**	1,36	1,44	2,98	4,71	10,28	0,77	8,16
**Cgi-Novel-19**	0,08	0,12	9,37	0,17	0,03	1,60	4,75
**Cgi-novel-10**	5,92	2,83	36,93	1,77	70,80	0,32	53,85
