# Supplementary figures and images for: Parallel analysis of miRNAs and mRNAs suggests distinct regulatory networks in Crassostrea gigas infected by Ostreid herpesvirus 1
Source: BMC Genomics. 2020 Sep 10;21:620. doi: 10.1186/s12864-020-07026-7 (PMC7488030; doi:10.1186/s12864-020-07026-7)

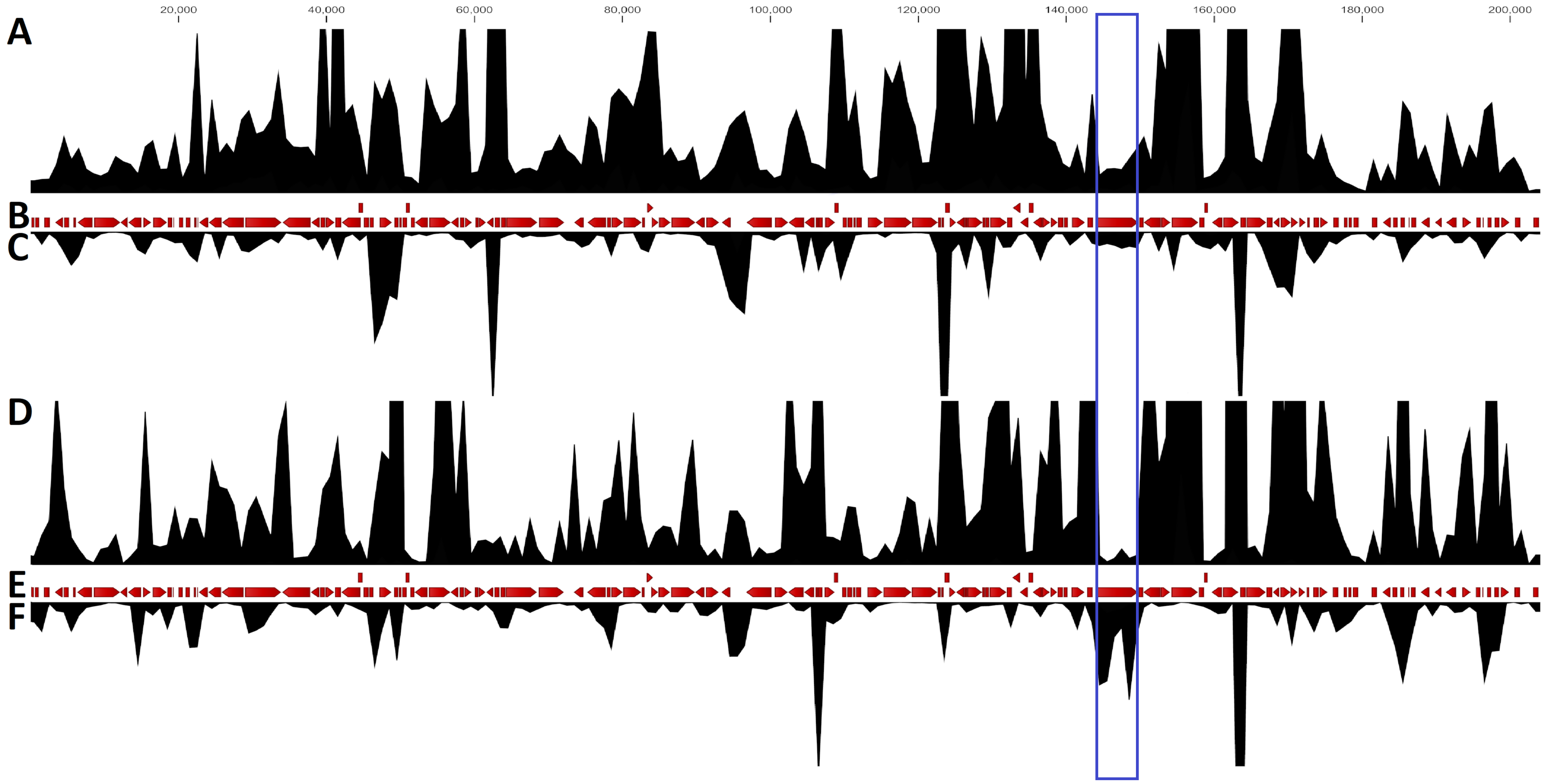

Supplement: Supplementary file 5 — Additional file 5. RNA-seq coverage of the OsHV-1 genome obtained with Ribo-depleted and polyA-selected libraries generated from sample S6. The coverage graph along the 204 kb OsHV-1 genome was reported in a 0-3000x scale for the Ribo-0 library mapped in sense direction (A), in antisense direction (C) and for the poly(A) library in sense direction (D) and antisense direction (F). The red arrows (B and E) depicted the OsHV-1 ORF annotations. The blue rectangle highlighted the viral DNA polymerase, ORF100. [file 12864_2020_7026_MOESM5_ESM.png]
