# Supplementary material for: Parallel analysis of miRNAs and mRNAs suggests distinct regulatory networks in Crassostrea gigas infected by Ostreid herpesvirus 1
Source: BMC Genomics. 2020 Sep 10;21:620. doi: 10.1186/s12864-020-07026-7 (PMC7488030; doi:10.1186/s12864-020-07026-7)
Supplement: Supplementary file 6 — Additional file 6. Oyster sampling data. [file 12864_2020_7026_MOESM6_ESM.pdf]

Additional File 5. Open-field challenge with *Crassostrea gigas* (Goro lagoon, late Spring 2016)

Figure 5.1 indicates the position of the lagoon of Goro relative to Venice (North Adriatic Sea, Italy).

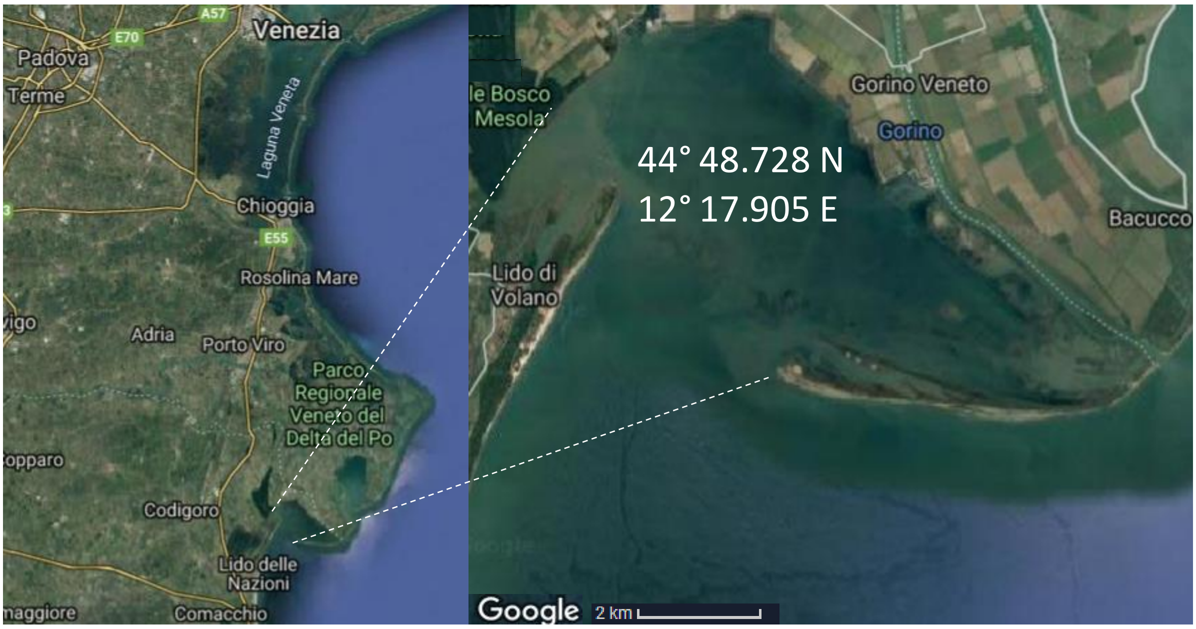

Figure 5.1. Left, coastal line defining the Venice lagoon and Po Delta lagoons in the North Adriatic Sea. Right, the coastal lagoon of Goro, named Sacca di Goro. Geographical coordinates indicate the *Crassostrea gigas* translocation site.

Following a work plan which allowed us to detect a OsHV-1-related infection and mortality of oyster spat in 2012 (Domeneghetti et al. 2014; doi:10.1016/j.fsi.2014.05.023) new *C. gigas* seed (3N, T6 size averaging 0.15 g, obtained from a French company) was positioned in Ostriga™ baskets to grow at 0.5-1 m depth in the Goro lagoon (44°48.728’N 12°17.905’E, North Adriatic Sea, Italy) on March 19, 2016. Subsequently, at least 30 oysters per sampling were regularly collected from Apr 26 to Jun 21 (26 April, 3-10-17-24-31 May, 7-14-21 June 2016).

No massive mortality was observed along the whole sampling period. The oyster spat reached a shell size of 2.46 ± 0.12 cm in about a month (17.05.2016) (**Figure 5.2**) and nearly doubled this size at the last sampling (4.78±0.25 cm; 21.06.2016). Native (diploid) oysters of similar size, farmed offshore the Goro lagoon area were also sampled for comparison (9.05.2016, 25.05.2016, 21.06.2016).

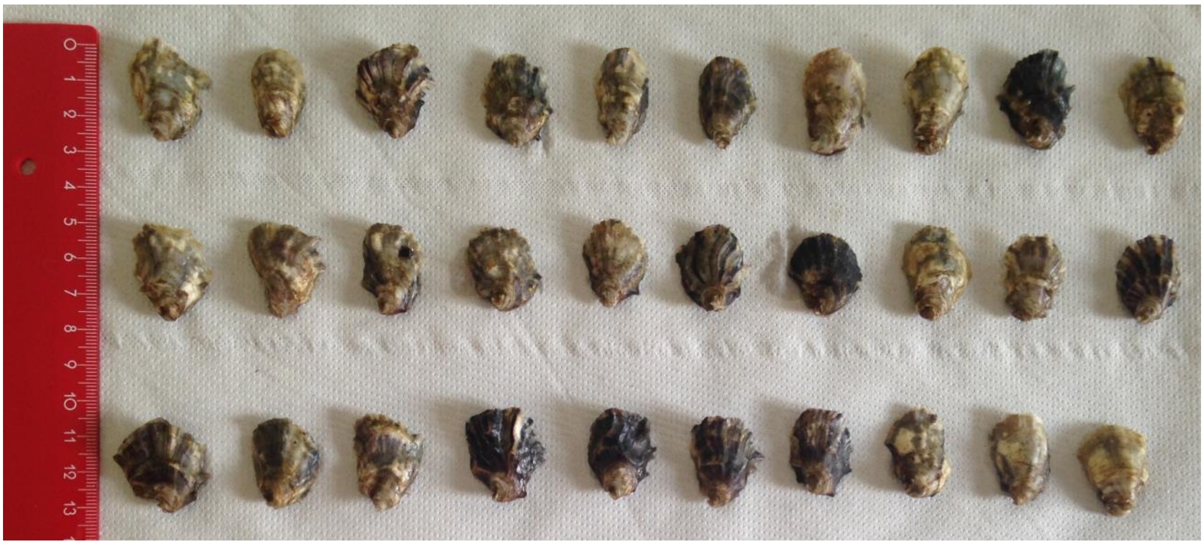

Figure 5.2. Size of the *Crassostrea gigas* oysters growing in the Goro lagoon at 17 May 2016

Following qRT-PCR analysis (Abbadi et al. 2018; doi:10.1099/jgv.0.001042), the oyster sampled on May 17 (2016) resulted to be variously positive for OsHV-1 DNA, with the amounts of viral DNA ranging from 0 to $1.8x10^9$ (mean and median values of 2.75 $x10^8$ and 1.1 $x10^8$, respectively). No OsHV-1 DNA was detected in the *C. gigas* oysters sampled offshore the Goro lagoon.

Temperature (°C), salinity (ppt) and dissolved oxygen (%) values hourly recorded in the sea water (SW) in close proximity to the deployed oyster spat, are reported below in order to illustrate the trends of key environmental factors in May 2016 and around the sampling of 17th of May 2016. Although irregularly fluctuating, the SW temperature increased from 15.7 °C to 25.1 °C in May 2016 (minimum and maximum values recorded on the 2nd and 28th of May, respectively) whereas SW salinity reached the minimum of 12.1 ppt on the 19th of May (**Figures 5.3 and 5.4**).

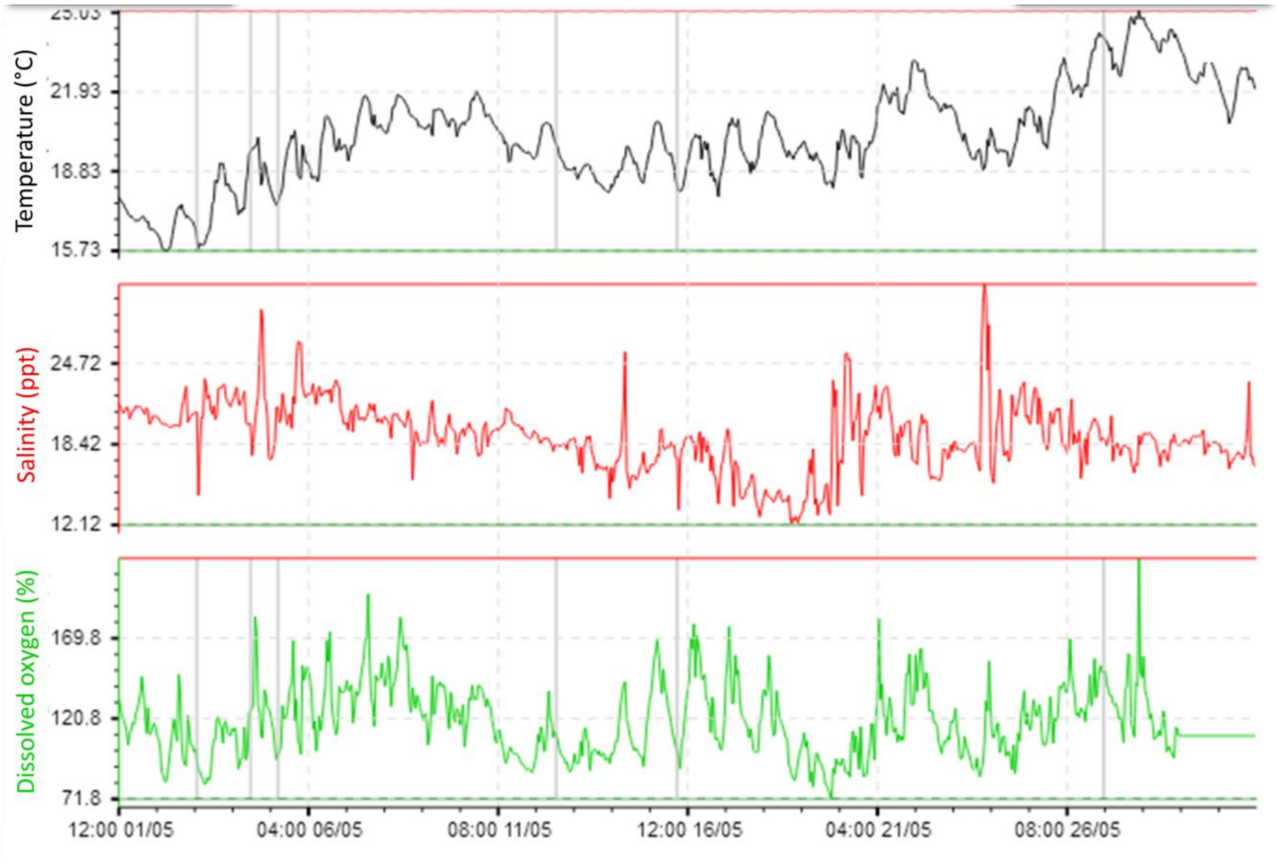

Figure 5.3. Trends of temperature (°C), salinity (ppt) and dissolved oxygen (%) in the Goro lagoon (from the top to the bottom, respectively) in the period 1-31 May 2016. Data records by courtesy of the Ferrara Province.

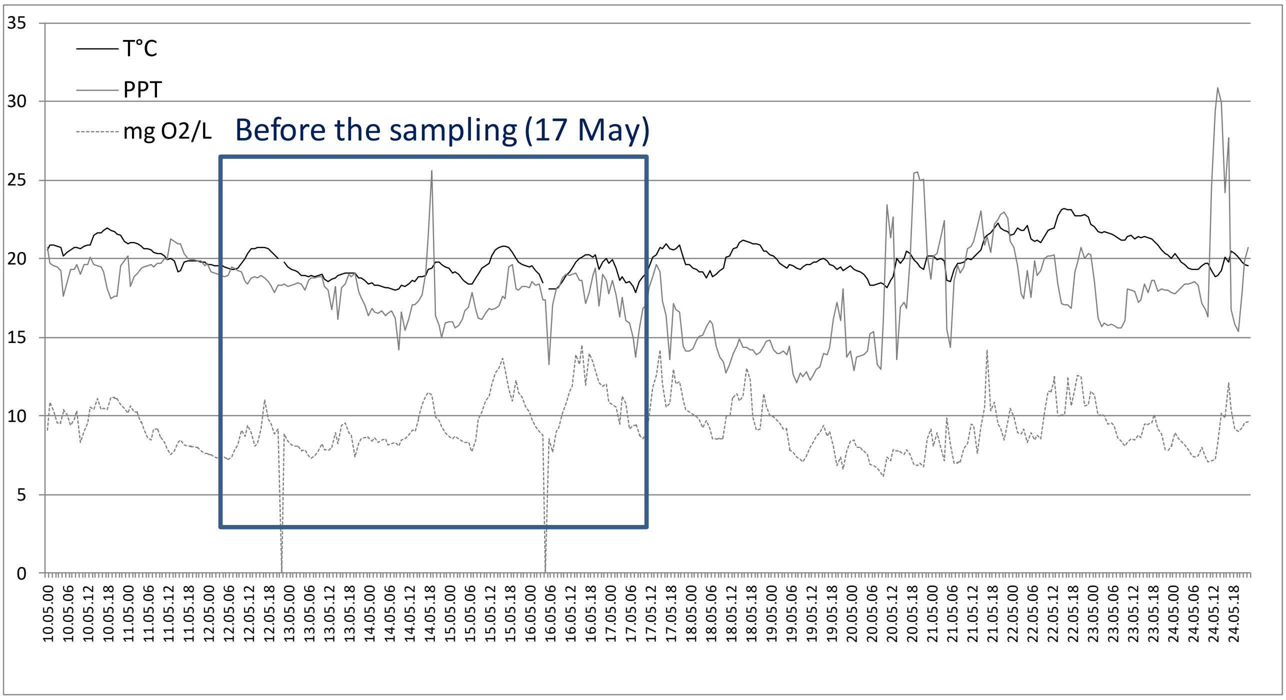

Figure 5.4. Trends of temperature (°C), salinity (ppt) and dissolved oxygen (mg/L) in the Goro lagoon in the period 10-24 May 2016 (*C- gigas* sampling occurred on the 17th of May 2019). Data records by courtesy of the Ferrara Province.

The ranges of temperature, salinity and dissolved oxygen recorded in the period 10-24 May 2019 (days around the sampling of oysters found OsHV-1 positive) are reported in Table S1.

Table S1. **Range of values recorded in the period 10-24 May 2016** (**Goro lagoon**)

	Lowest value	Highest value
**Temperature (°C)**	**17.8**	**23.6**
	[17/5]	[22/5]
**Salinity (ppt)**	**12.2**	**30.9**
	[19/5]	[24/5]
**Dissolved O_2 (mg/L)**	**6.12**	**14.5**
	[20/5]	[16/5]

The dates when these sea water data have been recorded are in square brakets.

In essence, the *C. gigas* spat sampled on 17.05.2019 were experiencing reduced salinity while the SW temperature was slowly but progressively increasing and dissolved oxygen widely fluctuating.
