# Supplementary material for: Parallel analysis of miRNAs and mRNAs suggests distinct regulatory networks in Crassostrea gigas infected by Ostreid herpesvirus 1
Source: BMC Genomics. 2020 Sep 10;21:620. doi: 10.1186/s12864-020-07026-7 (PMC7488030; doi:10.1186/s12864-020-07026-7)

**Additional File 6.** Comparison of two mRNA enrichment methods for dual RNA-seq analysis of *C. gigas* infected with OsHV-1.

RNA sequencing of the poly(A) and Ribo-0 libraries obtained from the sample S6 yielded 52 and 54 million of high-quality reads, corresponding to 7.1 and 6.7 Gb of sequencing readouts, respectively (Table 6.1). Strand-specificity was estimated to be 99.9 and 99.3% for the 2 libraries based on strand-specific mappings carried out on 7 oyster housekeeping genes, with no evidence of antisense transcription (Table 6.1). Read mapping on the oyster and OsHV-1 reference genomes showed that 96.8 and 78.4% of the poly(A) and Ribo-0 reads were on-target under the applied mapping conditions. The two libraries showed similar percentages of OsHV-1 reads, whereas the percentage of oyster reads was considerably reduced in the Ribo-0 library related to the poly(A) library (76.3 vs. 95%, Table 6.1). The *large gap read mapping* tool allowed the assignment of a small fraction of the unmapped reads to the oyster genome in both libraries (0.4 and 0.1%, respectively), but it did not recover additional viral reads (Table 6.1).

**Table 6.1.** High-throughput RNA sequencing of S6 and mapping results. Number of high-quality reads in millions, number of sequenced bases in billions, strand-specificity level and percentage of reads mapped on the *C. gigas* and OsHV-1 genomes using a standard and a *large gap read* (LGRM) mapping tools are reported as descriptors of the poly(A) selected and rRNA depleted libraries.

| Library | High quality reads [M] | Sequenced bases [Gb] | Strand Specificity (%) | % mapped (standard mapper) | | % mapped (LGRM) | |
|---|---|---|---|---|---|---|---|
| | | | | *C. gigas* | OsHV-1 | C. gigas | OsHV-1 |
| poly(A) | 51.98 | 7.1 | 99.9 | 95.0 | 1.8 | 0.4 | 0 |
| Ribo-0 | 54.14 | 6.7 | 99.3 | 76.3 | 2.1 | 0.1 | 0 |

The number of unmapped reads was different between the poly(A) and Ribo-0 libraries and the de-novo assembly of these reads resulted in 5,774 and 14,276 contigs, respectively, which were further classified into coding or putative non-coding transcripts. The Ribo-0 contigs showed a lower percentage of coding transcripts compared to those derived from the poly(A) library, while the percentage of putative lncRNAs was higher in the Ribo-0 contigs (Table 6.2).

**Table 6.2.** *De-novo* assembly of the poly A and Ribo-0 output reads. Number of unmapped reads in millions, number of assembled contigs and their classification into coding and putative non-coding transcripts are reported for the 2 libraries. A transcript was defined as ‘putative non-coding’ if it was longer than 500 bp and it did not encode viral ORFs.

| Library | Unmapped reads [M] | No. of de-novo contigs | % of coding | % of non-coding |
|---|---|---|---|---|
| poly(A) | 1.26 | 5,775 | 33 | 1.9 |
| Ribo-0 | 11.34 | 14,276 | 23 | 11.5 |

A total of 38 and 41% of the coding contigs of the poly(A) and Ribo-0 libraries, respectively, found a match in the NCBI nr-protein database (*blastx*, E-value cut-off $10^{-3}$). Most of these matches identified *C. gigas* or *C. virginica* hits, pinpointing a certain level of oyster inter-individual variability not absorbed by the reference genome. Several hits related to marine RNA viruses were also detected (Figure 6.1). Using the Rfam database, we identified 56 Ribo-0 hits similar to bacterial rRNA, while most of the putative non-coding contigs did not match to any known hit. The latter result can be explained with the higher aptitude of the Ribo-0 library to collect non-polyadenylated RNAs, including RNAs of bacterial origin. Moreover, we showed that the rRNA depletion procedure used to prepare the Ribo-0 library showed a

limited performance, since 17.5% of the reads matched to *C. gigas* rRNA sequences, compared to 0.3% in the poly(A) library.

**Figure 6.1. Summary of blast results.** Species assignment of the top-hit blast matches. A. Poly(A); B. Ribo-0

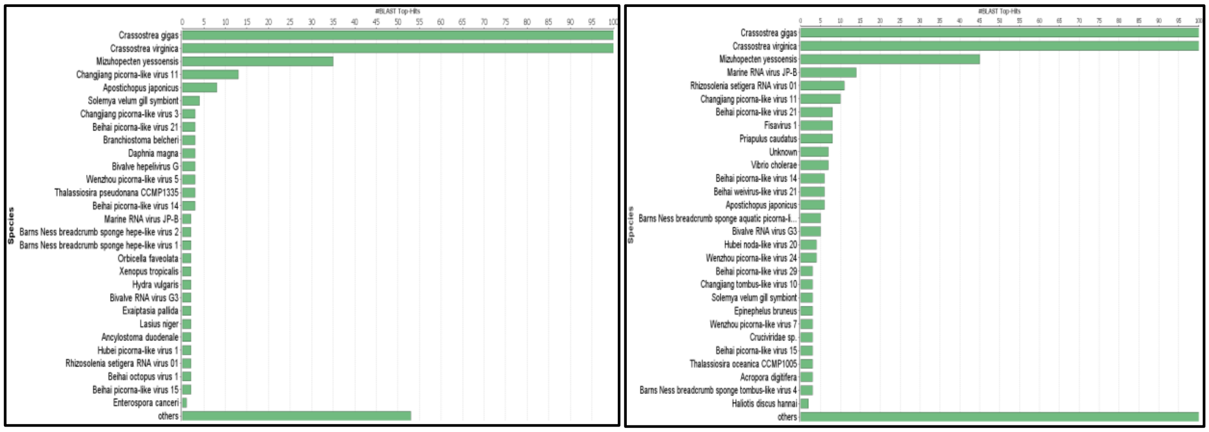

Supplement: Supplementary file 7 — Additional file 7 Comparison of two mRNA enrichment methods for dual RNA-seq analysis of C. gigas infected with OsHV-1. [file 12864_2020_7026_MOESM7_ESM.pdf]
